# Effect of Different Magnetite Nanoparticle Coatings on Blood Circulation, Biodistribution, Tumor Accumulation and Penetration

**DOI:** 10.3390/pharmaceutics18030345

**Published:** 2026-03-11

**Authors:** Elizaveta N. Mochalova, Maria A. Yurchenko, Tatiana S. Vorobeva, Darina A. Maedi, Nikita O. Chernov, Olga A. Kolesnikova, Ekaterina D. Tereshina, Victoria O. Shipunova, Maria N. Yakovtseva, Petr I. Nikitin, Maxim P. Nikitin

**Affiliations:** 1 Moscow Center for Advanced Studies, 20 Kulakova St, 123592 Moscow, Russia; 2 Department of Nanobiomedicine, Sirius University of Science and Technology, 1 Olimpiyskiy Ave, 354340 Sirius, Krasnodar Region, Russia; 3 Moscow Engineering Physics Institute, 31 Kashirskoe Shosse, 115409 Moscow, Russia; 4 Shemyakin-Ovchinnikov Institute of Bioorganic Chemistry, Russian Academy of Sciences, 16/10 Miklukho-Maklaya St, 117997 Moscow, Russia

**Keywords:** magnetic nanoparticles, magnetite, iron oxide nanoparticles, polymers, lectins, blood circulation time, pharmacokinetics, biodistribution, MRI, targeted drug delivery

## Abstract

**Background/Objectives:** Magnetite nanoparticles represent promising candidates for a broad spectrum of biomedical applications, ranging from in vitro diagnostic assays to in vivo imaging, hyperthermia, and targeted drug and gene delivery, with some nanoagents already approved for clinical use. A critical determinant of their functionality is the nanoparticle coating, which facilitates beneficial interactions within biological systems. In the context of tumor-targeted therapeutic delivery, key design parameters—particularly surface coatings—can be optimized to enhance treatment efficacy by modulating blood circulation kinetics, biodistribution, and other critical properties. However, current preclinical screening methods primarily rely on cell culture models to identify potential nanocarriers, yet these systems often poorly correlate with actual in vivo performance. This discrepancy highlights the necessity of incorporating more biologically relevant testing platforms, such as high-throughput in vivo assays. **Methods:** In this work, we employed an original magnetic particle quantification (MPQ) technology to systematically evaluate the blood circulation kinetics and biodistribution patterns for magnetite nanoparticles with 17 different coatings across multiple organs and tissues, including the liver, spleen, lungs, kidneys, heart, tumor, brain, peripheral blood, muscle, and bone. This methodology offers high sensitivity, user-friendly operation, and provides quantitative measurements across a broad dynamic range of nanoparticle concentrations. These advantages enabled high-throughput acquisition of precise blood circulation and biodistribution data. In addition, histological analysis was conducted to evaluate nanoparticle penetration depth within tumor tissue. **Results:** Here we conducted a comprehensive study of the effect of 17 different polymer-, lectin-, and small molecule-based coatings on the behavior of magnetite nanoparticles in vivo. For each type of obtained nanoparticles, we implemented passive targeting as well as magnetic targeting, the latter using an external magnetic field localized in the tumor area. **Conclusions:** The collected dataset provides critical insights into how surface modifications influence nanoparticle performance in complex biological systems, offering valuable guidance for optimizing therapeutic nanocarrier design.

## 1. Introduction

Superparamagnetic iron oxide nanoparticles (SPIONs) are widely used in biotechnology and medicine due to their magnetic properties and high biocompatibility [1,2]. These nanoparticles can be precisely controlled by an external magnetic field [3], enabling targeted drug delivery and intracellular hyperthermia for cancer therapy [4]. SPIONs usually include two types of nanomaterials: magnetite (Fe_3_O_4_) and maghemite (γ-Fe_2_O_3_) [5]. In practice, magnetite-based nanoparticles tend to be the preferred option for biomedical applications: as contrast enhancers for magnetic resonance imaging (MRI) [6], as carriers for targeted drug delivery and photodynamic therapy [7], and for isolating cells from peripheral blood or other tissues, which is important for cancer diagnosis, therapy monitoring, and drug development [8,9,10].

Some magnetite nanoparticles have already been approved by the Food and Drug Administration (FDA) [11], for example, as contrast agents for MRI (Lumirem (EU), GastroMARK (USA)) and therapeutic agents for the treatment of iron deficiency anemia (Feraheme (USA)) [12,13]. In addition, the vast majority of magnetite nanoparticles are in preclinical trials [14].

However, the use of this promising nanomaterial for tumor targeting remains limited due to nanoparticle aggregation tendencies and short blood circulation times caused by rapid clearance via the mononuclear phagocyte system, primarily by Kupffer cells in the liver and tissue macrophages of the spleen [15,16]. Consequently, this rapid clearance directly compromises nanoparticle delivery efficiency to tumors.

To address these and other issues, nanoparticles are typically coated with a wide range of materials [15,17]. Among them, the most widely used are various biocompatible polymers. In order to extend the circulation time of nanoparticles and increase their therapeutic effectiveness, the surface of nanoparticles is modified with polyethylene glycol (PEG). This polymer reduces the non-specific interaction of nanoparticles with blood proteins and thus “hides” them from recognition by the immune system [18]. The surface can also be modified with other polymers (such as chitosan, dextran, etc.) to stabilize the nanoparticles either sterically or by providing a sufficient surface charge. In addition, some functional groups of polymers can be used for further modification of nanoparticles with drugs, fluorescent labels, and receptors (e.g., antibodies, lectins, etc.), recognizing various biological targets in order to create complex systems for targeted drug delivery to tumors and other organs and tissues [19,20,21,22]. In the case of magnetite nanoparticles, polymer coatings can also prevent their oxidation to maghemite [23].

Nanoparticles can also be coated with lectins—glycoproteins that selectively bind to carbohydrates and participate in intercellular recognition processes, host–pathogen interactions, immune regulation, etc. [24]. Lectins were first discovered and isolated from plants and have been widely used in biomedical practice due to their ability to specifically recognize and bind both dissolved carbohydrates and their functional groups in glycoproteins and glycolipids. The development of malignant tumors is accompanied by the overexpression of certain oligosaccharides (e.g., fucose and sialic acid) on the cell surface, which makes it possible to use lectins for targeted cancer treatment [25]. They are also used in diagnostic test systems for detecting cancer cells [26]. Thus, polymer- and lectin-based coatings may be a key factor determining the circulation kinetics and biodistribution profile of nanoparticles, as well as their ability to penetrate tumor tissues.

Previously, we implemented a method for high-precision analysis of the kinetics and biodistribution of magnetic particles—magnetic particle quantification (MPQ) technology, which allows quantitative and real-time determination of the content of nonlinear magnetic materials in the bloodstream, organs, and tissues. The method demonstrates high sensitivity and selectivity. It has a detection limit of 0.4 ng of nanoparticles in a 200 µL volume (60 zmol) [27] and is selective for nanoparticle detection, as neither biological tissues nor endogenous iron contributes to the MPQ signal [28]. MPQ is actively used in the field of diagnostics—for studying the interactions of magnetic particles with cells, as well as for the rapid detection of toxins, markers of various diseases, hormones, and extracellular vesicles [29].

In this work, we investigated the influence of different surface coatings on the in vivo behavior of magnetite nanoparticles (NPs). For each type of obtained NPs, we implemented both passive targeting and magnetic targeting—the latter guided by an externally applied magnetic field focused on the tumor site. Using the highly sensitive MPQ method, we obtained quantitative data on the circulation of nanoparticles in the bloodstream, their biodistribution, and tumor accumulation efficiency. In addition, we performed a histological analysis to assess the penetration depth of nanoparticles into tumor tissues. The obtained data will be useful for the creation of therapeutic systems based on nanomaterials, in particular magnetite NPs, with improved efficiency and safety.

## 2. Materials and Methods

### 2.1. Materials

The following reagents were used in the experiments: iron(III) chloride hexahydrate (Sigma-Aldrich, St. Louis, MO, USA); iron(II) chloride tetrahydrate (Sigma-Aldrich, USA); Zoletil 100 (Virbac, Carros, Provence, France); Xyla (Interchemie Werken De Adelaar Eesti AS, Harju County, Püünsi, Estonia); carboxymethyl-dextran sodium salt (4, 40, 70, 150 kDa) (Carbosynth, Compton, Berkshire, UK); carboxymethyl-dextran sodium salt (BioXtra, Sigma-Aldrich, USA); chitosan oligosaccharide lactate 5 kDa (Sigma-Aldrich, USA); dextran from *Leuconostoc* spp. (Sigma-Aldrich, USA); polyethylene glycol 6 kDa (Fluka Analytical, Darmstadt, Germany); polyethylenimine branched (Sigma-Aldrich, USA); poly(vinyl alcohol) 13–23 kDa (Sigma-Aldrich, USA); poly(acrylic acid) 70 kDa (Sigma-Aldrich, USA); poly(N-vinyl-pirrolidone) 8 kDa (Alfa Aesar, Ward Hill, MA, USA); sodium polystyrene sulfonate (70, 500 kDa) (Alfa Aesar, USA); tri-sodium citrate 2-hydrate (PanReac AppliChem, Barcelona, Spain); concanavalin A (Sigma-Aldrich, USA); soybean agglutinin (Vector Laboratories, Newark, CA, USA); 1-ethyl-3-(3-dimethylaminopropyl)carbodiimide (EDC) (Sigma-Aldrich, USA); N-hydroxysulfosuccinimide (sulfo-NHS) (Carbosynth, UK); 2-(N-morpholino)ethanesulfonic acid sodium salt (MES); HEPES (Diaem, Moscow, Russia); tris(hydroxymethyl)aminomethane hydrochloride (Tris-HCl) (Diaem, Russia); potassium chloride (KCl) (PanReac AppliChem, Spain); potassium hexacyanoferrate(II) 99% (Kemstor, Moscow, Russia); RPMI-1640 essential media (PanEco, Moscow, Russia); fetal bovine serum (Hyclone, Logan, UT, USA); penicillin-streptomycin (PanEco, Russia); L-glutamine (PanEco, Russia); gentamicin (PanEco, Russia); hydrochloric acid (Khimmed, Moscow, Russia); eosin (Biovitrum, St. Petersburg, Russia); Harris hematoxylin (Biovitrum, Russia); ethanol 95%; methanol 99%; xylene; PBS buffer (Fisher Scientific, Waltham, MA, USA; PBS, pH 7.4); D(+)-glucose anhydrous (Panrec Applichem, Spain); glycogen from oyster (Sigma-Aldrich, USA); mucin from porcine stomach Type III (Sigma-Aldrich, USA); D-(+)-galactose (Sigma-Aldrich, USA); bovine serum albumin (PanEco, Russia); Milli-Q water (Merck Millipore, Darmstadt, Germany) was used for the preparation of aqueous solutions.

### 2.2. Cell Culture

The murine colon carcinoma cell line CT26 was maintained in RPMI-1640 medium supplemented with 10% fetal bovine serum, 50 units/mL penicillin-streptomycin, 10 mg/L gentamicin, and 300 mg/L L-glutamine. Cells were incubated at 37 °C in a 5% CO_2_ atmosphere and passaged routinely upon reaching 80–90% confluence. Cell counts were determined using a Luna-II automated cell counter (Logos Biosystems, Anyang-si, Gyeonggi-do, Republic of Korea). This procedure involved mixing cell suspensions 1:1 with 0.4% trypan blue prior to loading onto counting slides.

### 2.3. Animals

All experimental procedures were approved by the Institutional Animal Care and Use Committee of the Shemyakin-Ovchinnikov Institute of Bioorganic Chemistry, Russian Academy of Sciences, according to protocol # 375/2023 (20 September 2023–19 September 2026). Mice were group-housed in a standard vivarium under controlled conditions (12/12 h light/dark cycle, 22 ± 2 °C) with ad libitum access to food and water. Animals were acclimatized to vivarium conditions for at least two weeks prior to any experimental procedures. Female and male BALB/c mice (2–4 months old, 18–25 g) were used in the study. The animals were anesthetized via intraperitoneal injection of a Zoletil 100/Xyla combination at a dose of 40/1.5 mg/kg. To establish tumor-bearing mice, 2 × 10^6^ CT26 cells in 200 µL of serum-free culture medium were inoculated subcutaneously into the right flank. Tumor size was monitored by caliper measurement, and volume (V) was calculated using the formula: V = (width^2^ × length × 3.14)/6.

### 2.4. Synthesis of Magnetite Nanoparticles

NPs were synthesized using the microemulsion method described earlier [30]. A total of 2 g of sodium dodecyl sulfate (SDS) was dissolved in a mixture of 20 mL of n-hexane and 12 mL of n-butanol and heated for 20 min at 40 °C. It should be noted that complete dissolution of SDS does not occur at this stage due to the absence of water in the reaction mixture. Then, 0.083 g of FeSO_4_∙7H_2_O dissolved in 0.6 mL of MilliQ water and 0.135 g of FeCl_3_∙6H_2_O dissolved in 1 mL of MilliQ water were added with a 2 min interval to the mixture until an orange transparent solution was obtained. The resulting mixture was heated for 20 min at 40 °C, and then the heating temperature was increased to 70 °C, and 2 mL of 25% NH_3_ solution was added quickly using a syringe, whereupon the solution acquired a black color. Then the nanoparticle solution was incubated at 70 °C for 40–60 min, after which it was cooled to room temperature. The obtained magnetic NPs were washed sequentially three times with 96% EtOH and water.

### 2.5. Analysis of Magnetite Nanoparticles by SEM and TEM

Scanning electron microscopy (SEM) images were obtained on a Brucker Crossbeam 550 (Brucker, Karlsruhe, Germany) microscope. Colloidal samples were applied to a silicon wafer and dried at room temperature. Transmission electron microscopy (TEM) images were obtained on a JEM-2100plus (JEOL, Akishima, Japan) microscope at an accelerating voltage of 200 kV. To obtain the size distribution profile, TEM images were analyzed using the ImageJ 1.54g program (at least 100 NPs were measured).

To prepare samples after incubation in serum, 1 mL of mouse blood was collected, incubated at room temperature for 30 min to allow clot formation, then centrifuged at 500× *g* for 15 min to remove the clot, and finally at 15,000× *g* for 5 min to remove large molecular aggregates. A total of 50 µL of nanoparticle suspension was mixed with 50 µL of serum and incubated for 10 min. Subsequently, the mixture was washed thoroughly using four cycles of centrifugation (15,000× *g*, 5 min) and resuspended in deionized water to remove excess serum proteins.

### 2.6. Modification of Magnetite Nanoparticles with Various Coatings

To modify the NPs with carboxymethyldextran 4 kDa, 12 mg of magnetite NPs in 100 µL of water were mixed with 100 mg of polymer in 300 µL of MilliQ water. The resulting solution was placed in an ultrasonic bath for a few seconds and then incubated in a water bath thermostat at 90 °C for 5 min. After that, the NPs were cooled at 4 °C for another 5 min. This procedure was repeated three times, and the mixture was left to incubate overnight at room temperature. The coated NPs were centrifuged for 10 min at 10,000× *g*, and then washed three times with 2 mL of MilliQ water by centrifugation in order to remove unbound polymer. Coating with other polymers was performed in the same way as described above (the amounts of NPs and coatings used in the modification are listed in Table 1, the detailed coating protocols are summarized in Table S1).

To modify NPs with lectins, the carbodiimide conjugation method was used. 1 mg of (CMD 10–20 kDa)@NPs in 15 µL of water was mixed with 85 µL of 0.1 M MES buffer (pH 5.0), containing 2 mg of EDC and 1 mg of sulfo-NHS. The resulting mixture was incubated for 20 min at room temperature. Then, the NPs were washed from unbound crosslinkers by centrifugation for 10 min at 10,000× *g*. After that, 200 µL of a lectin solution at a concentration of 0.5 g/L in 0.1 M HEPES buffer (pH 6.0) was added to the particles and sonicated for 5 s. Finally, the NPs were incubated at 4–10 °C overnight and washed three times to remove unbound lectins by centrifugation.

### 2.7. Measurements of Hydrodynamic Size and Zeta Potential of Coated Magnetite Nanoparticles

The hydrodynamic sizes of nanoparticles with different coatings were determined using a BeNano 180 Zeta Pro (Bettersize, Dandong, Liaoning, China) in the following solutions: MilliQ water, 10 mM acetate buffer (pH 5), 10 mM acetate buffer (pH 5) with 150 mM NaCl, 10 mM Tris-HCl buffer (pH 9), and 10 mM Tris-HCl buffer (pH 9) with 150 mM NaCl. Zeta potential measurements were performed in 10 mM acetate buffer (pH 5), Tris-HCl buffer with KCl (1 mM Tris, 2 mM KCl, pH 7), and 10 mM Tris-HCl buffer (pH 9). For all measurements, 20 µL of 0.2% nanoparticle solution was mixed with 1 mL of the corresponding buffer. All experiments were performed in *n* = 3 technical replicates.

For size measurements in serum, 10 µL of a nanoparticle suspension at 10 g/L was mixed with 40 µL of serum (this ratio ensures protein adsorption saturation [31]), and 5 µL of the mixture was placed into a thin capillary cuvette for DLS measurements at a 90° scattering angle. For zeta potential, 45 µL of the nanoparticle–serum mixture was diluted to 1 mL with PBS to maintain native ionic strength, after which the zeta potential was determined using electrophoretic light scattering.

### 2.8. Measurement of Coated Magnetite Nanoparticle Circulation Kinetics and Circulation Half-Life in the Bloodstream of Mice

To study the kinetics of nanoparticle circulation, the tail of the anesthetized mouse was placed in the coil of the MPQ device, then NPs at a dose of 1 mg in 100 µL of 5% glucose were injected i.v. into the retro-orbital sinus. The magnetic signal was recorded every 1.6 s until it completely dropped to the noise level. To determine the circulation half-life of the nanoparticles, we plotted the magnetic signal, normalized to the maximum, versus time. The signal values in the range from 0.9 to 0.1 were approximated by the curve y = a*e*^*bx*^. Then, from the values of the coefficients, the half-life was calculated using the formula $t_{1/2}=ln2/(-b)$.

### 2.9. Study of Biodistribution and Accumulation in Tumors

To quantitatively assess the biodistribution of coated and uncoated magnetite nanoparticles in tumor-bearing mice, two experimental groups were established. The first group received a retro-orbital injection of 1 mg of nanoparticles. Twenty-four hours post-injection, the mice were sacrificed via cervical dislocation, and the following organs and tissues were harvested: heart, lungs, liver, spleen, kidneys, tumor, brain, muscle, bone, and blood. For magnetic signal quantification, 50 mg samples from each organ or tissue were placed in the coil of an MPQ device. The integral magnetic signal from each sample was normalized to the total signal obtained from all measured organs and tissues to determine relative nanoparticle accumulation. The second group of mice underwent the same nanoparticle administration procedure, but in this case, a neodymium magnet (20 mm × 10 mm × 5 mm) was applied to the tumor area for 3 h to achieve magnetic targeting. The magnetic field induction of the magnet as a function of the distance from its surface is presented in Supplementary Note S1 and Figure S1.

### 2.10. Histological Analysis

A part of the tumor (see Section 2.9 of Materials and Methods) was frozen at −80 °C immediately after extraction. Not earlier than several hours later, the tissues were cut into 7 µm-thick sections on a Cryotome FSE cryostat (Thermo Scientific, Waltham, MA, USA). For staining, tumor samples were placed in a 10% potassium hexacyanidoferrate(II) solution for 30 min, then in a 1:1 solution of 10% potassium hexacyanidoferrate(II) and 20% HCl for another 5 min. The reaction was stopped by placing the sample in water. The tissues were then placed in a hematoxylin solution for 1.5 min, washed with water, and placed in an eosin solution for 15 s. Fixation was performed with 95% ethanol for 10 s, then the sample was alternately placed in methanol and xylene for 10 s each. The preparation was coated with a mounting medium and covered with a cover glass, after which it was left to dry overnight. The samples were then examined on an Olympus Fluoview FV3000 confocal microscope (Olympus Optical Co., Ltd., Tokyo, Japan) in bright field mode. Using ImageJ software, the depth of nanoparticle penetration into the tumor tissue was determined on the acquired images by measuring the perpendicular distance from the 10 most distant detected nanoparticles to the edge of the section [32].

### 2.11. Magnetic Resonance Imaging

BALB/c mice were intravenously injected with 1 mg NP in 100 µL 5% glucose. NPs were focused on the tumor area by an external magnetic field using a 20 mm × 10 mm × 5 mm neodymium magnet. After 3 h of nanoparticle circulation, mice were sacrificed and imaged using an ICON 1T MRI system (Bruker, Karlsruhe, Germany) using a whole-body radiofrequency coil. Uninjected BALB/c mice were used as a control. We used a FLASH sequence with the following parameters: repetition time—1000 ms, echo time—5 ms, flip angle—60 degrees, field of view—8/5 cm, 17 slices per scan, slice thickness—1 mm.

### 2.12. Statistical Analysis

All in vivo experiments were conducted with the sample size (*n*) specified for each test group in the corresponding figure. Statistical analyses were carried out using Microsoft Excel version 2016 and GraphPad Prism 8.0.1. Data are presented as bar graphs displaying mean values ± standard deviation. To evaluate differences, one-way ANOVA followed by Dunnett’s multiple comparisons test was applied, and two-way ANOVA followed by Sidak’s multiple comparisons test was used to compare magnetic versus passive targeting for each nanoparticle type. Additionally, Welch’s *t*-test was performed. The full statistical results are available in the file uploaded to the Figshare repository: https://doi.org/10.6084/m9.figshare.31361680 (accessed on 5 March 2026). Statistical significance was defined as follows: ns—*p* > 0.05; *—*p* ≤ 0.05; **—*p* ≤ 0.01; ***—*p* ≤ 0.001.

### 2.13. Use of Artificial Intelligence (AI) Tools

In preparing this manuscript, the authors used DeepSeek 3.0 exclusively for translation from their native language into English. After using the tool, the authors reviewed and edited the content and took full responsibility for the final version of the publication.

## 3. Results and Discussion

### 3.1. Synthesis of Magnetite Nanoparticles

First, we synthesized magnetite NPs using the water-in-oil microemulsion method as described previously [30]. Briefly, this method involves the use of an emulsion system containing aqueous and oil phases and a surfactant to stabilize the interphase boundary. Ferric and ferrous salts were dissolved in the water phase, where magnetite nanoparticles were formed after ammonia addition. The emulsion was heated, and then the resulting NPs were isolated from the mixture by magnetic separation and washed sequentially with ethanol and water.

**Figure 1:**
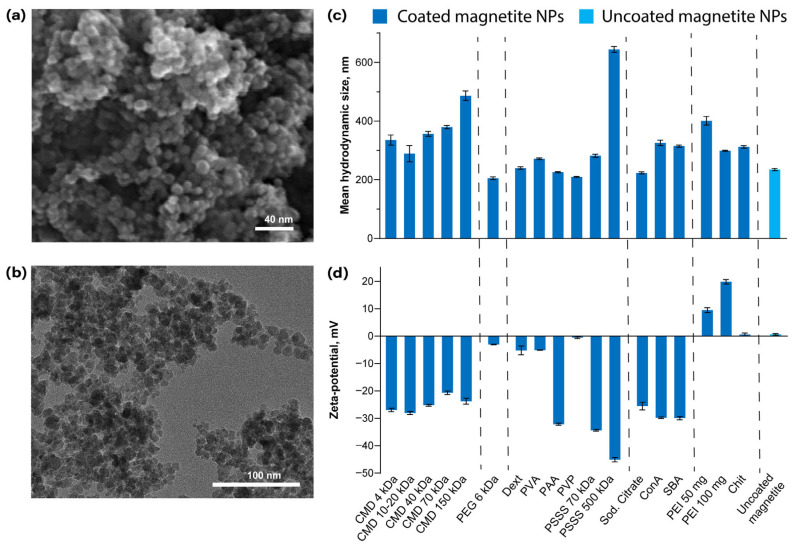
Characterization of magnetite nanoparticles: SEM (**a**) and TEM (**b**) images of uncoated magnetite nanoparticles; (**c**) Mean hydrodynamic size in MilliQ water; (**d**) Zeta potential at pH 7. The DLS data are presented as mean values of individual measurements ± standard deviations, *n* = 3. Dashed lines are used to visually align the names of the coatings with their corresponding bars.

The morphology and size of the obtained NPs were studied using scanning electron microscopy and transmission electron microscopy (Figure 1a,b). The average size of NPs according to TEM results was 19 ± 4 nm, which is consistent with the data described in the literature [33,34].

### 3.2. Surface Modification of Magnetite Nanoparticles with Various Coatings

Table 1 presents 18 variants of surface coatings of magnetite nanoparticles studied in this work. We used compounds of three classes as coatings: polymers, lectins, and a small molecule. These coatings differ from each other in their physicochemical properties and are actively used to modify nanoparticles in various biomedical studies. All the nanoparticle coatings were introduced as described in detail in the Materials and Methods Section.

Dextran is a polysaccharide that is a branched polymer of glucose and is synthesized by some lactic acid bacteria. It has good biocompatibility, biodegradability, and hydrophilicity. Dextran is used as an anticoagulant, to reduce blood viscosity, and for the emergency treatment of hypovolemia. The antithrombotic effect of dextran is due to its ability to bind to blood cells and vascular endothelium, thereby increasing their negative surface charge and reducing thrombus formation [35]. Dextran also serves as a basis for drug delivery systems [36]. It is used as a coating for NPs to stabilize them and increase their bioavailability [37]. In our study, dextran with a chain mass of 70 kDa was used.

Carboxymethyldextran (CMD) is a dextran derivative in which some hydrogens of the hydroxyl groups are replaced by carboxymethyl groups. In this work, we used several types of carboxymethyldextran with average chain weights of 4, 10–20, 40, 70, and 150 kDa. The presence of carboxyl groups allows not only to stabilize nanoparticles from aggregation due to both steric and electrostatic stabilization factors, but also to use well-studied protocols for covalent immobilization (e.g., carbodiimide conjugation) of enzymes, antibodies, peptides, lectins, or low molecular weight compounds [38,39]. Such functionalization of nanoparticles enables the creation of complex biocomputing interfaces on their surface, sensitive to various molecules in the microenvironment [2,40].

Chitosan is a biocompatible and biodegradable linear polysaccharide obtained from the chitin of crustacean shells by its deacetylation. Some chitosan properties, such as antibacterial activity and mucoadhesive ability, are due to its cationic nature [41]. Also, the presence of –NH_2_ and –OH groups in the structure of chitosan allows it to be modified to increase solubility in water and organic solvents, sensitivity to pH, and change other physicochemical properties. Various modifications of chitosan have many biomedical and commercial applications: as targeted delivery systems [42], as antimicrobial agents [43], and even for the creation of rechargeable water batteries [44]. In the field of nanotechnology, chitosan is used both alone and as a coating for other types of NPs [45]. Here, we used positively charged chitosan with an average chain mass of 5 kDa.

As we noted earlier, coating nanoparticles with polyethylene glycol is one of the effective ways to prevent the adsorption of proteins onto the surface of the nanomaterial, thereby impeding its recognition by immune cells and thus leading to an extension of their circulation in the bloodstream [46]. This type of coating is widely used in clinical practice, for example, in the composition of PEGylated liposomal doxorubicin, known by the names of Doxil^®^ (USA), Lipodox^®^ (generic Doxil), and Caelyx^®^ (Europe) [27]. For PEGylating magnetite nanoparticles, we used PEG with an average chain weight of 6 kDa.

Polyvinyl alcohol (PVA) is a synthetic polymer that is water-soluble. Polyacrylic acid (PAA) is a polymer of acrylic acid with anionic properties at neutral pH, which is used for nanoparticle coatings, for example, to employ them in heavy metal adsorption [47]. Polyvinylpyrrolidone (PVP) is a water-soluble polymer consisting of N-vinylpyrrolidone monomers. It has low toxicity and is biocompatible. These polymers are FDA approved for use in medicine and cosmetology, in particular, in the composition of slow-release tablets, dressing materials, and as thickeners in shampoos, adhesives, latex, etc. [48].

Sodium polystyrene sulfonate (PSSS) is an ion-exchange resin structurally composed of a polystyrene backbone modified with sulfonate groups. In medicine, PSSS acts as a potassium-binding agent in hyperkalemia therapy, releasing sodium ions in exchange. Additionally, PSSS-based polyelectrolyte hydrogel membranes serve for controlled drug delivery [49]. In other fields, it is used in emulsifiers, dispersants, cleaning agents, and heat-transfer products. In this work, we employed PSSS with average chain weights of 70 and 500 kDa.

Polymerization product of aziridine, polyethyleneimine (PEI), has a number of applications in biomedicine due to its cationic nature, such as promoting cell culture adhesion to the bottom of culture flasks [50]. PEI is widely used as a gene delivery vehicle due to the following aspects: it promotes DNA condensation, protecting it from nucleases and promoting its endocytosis into cells; and it facilitates the release of DNA from cellular lysosomes via the proton sponge mechanism [51,52]. PEI coating has also demonstrated high efficiency in delivering siRNA into cells [53].

Sodium citrate is used in food production as a preservative and in medicine as an anticoagulant due to its ability to bind calcium ions, thereby preventing blood clotting. Coating nanoparticles with sodium citrate provides them with a negative zeta potential, which promotes their mutual repulsion and, as a result, leads to a decrease in aggregation [54].

In this study, we also used two types of lectins as coatings for NPs: soybean agglutinin (SBA) and concanavalin A (ConA). Con A, obtained from *Canavalia ensiformis* plant, specifically binds to glycoproteins containing mannose and glucose residues, while not binding to galactose, fucose, and sialic acid. It also has mitogenic activity, stimulating the proliferation and differentiation of T-lymphocytes [55]. ConA is used to purify glycosylated molecules by lectin affinity chromatography [56], to create biosensors that monitor blood glucose levels [57] and to characterize sugar-containing substances and cells [58]. ConA has antiproliferative and antitumor activities, making it attractive as a potential agent for cancer therapy [59].

SBA is a lectin found in soybeans that preferentially binds to N-acetylgalactosamine or galactose [60]. SBA is an antinutrient due to its ability to bind specifically to intestinal epithelial cells, causing inflammation. In biomedicine, SBA is used to select hematopoietic stem cells and as a T-cell depleting agent, which ultimately reduces graft rejection in bone marrow transplants [61]. SBA has also been used as a prognostic indicator in gastric cancer research [62].

After modifying the nanoparticles with the above coatings, their hydrodynamic size and zeta potential were measured using dynamic light scattering (DLS) in order to confirm polymer coating and investigate nanoparticles’ colloidal stability (Figure 1c,d). As expected, the surface charge of the nanoparticles at pH 7 varied depending on the coating. Uncoated magnetite exhibited near-zero zeta potential since its isoelectric point (pH_iep_) is close to 7 [63]. (PEI 50 mg)@NPs and (PEI 100 mg)@NPs showed positive zeta potentials, consistent with PEI’s high pH_iep_ (10.5–11) due to its polycationic nature, because of -NH_2_ groups protonation [64]. Chit@NPs, despite chitosan being cationic, displayed near-neutral charge at pH 7, which aligns with chitosan’s relatively low pH_iep_ (7.5–8) [65]. PAA@NPs, (PSSS 70 kDa)@NPs, (PSSS 500 kDa)@NPs, and all CMD-coated nanoparticles exhibited strongly negative zeta potentials due to proton dissociation from -COOH and -SO_3_H groups in these polymers. Regarding PVA@NPs, PVP@NPs, (PEG 6 kDa)@NPs, and Dext@NPs, these particles showed near-zero zeta potentials in neutral conditions due to the absence of protonatable or deprotonatable functional groups in these polymers’ structures.

One can note that nanoparticle sizes measured in MilliQ water show no correlation with zeta potential values. Indeed, under very low ionic strength conditions, even uncoated magnetite nanoparticles remain temporarily stable (exhibiting hydrodynamic diameters close to 200 nm), indicating that zeta potential has minimal influence on size under these conditions. Most of the coated nanoparticles show hydrodynamic diameter values in the 200–300 nm range, suggesting they exist in solution as aggregates of similar size to uncoated magnetite. Exceptions include many of the CMD-coated particles (particularly (CMD 150 kDa)@NPs), (PSSS 500 kDa)@NPs, and (PEI 50 mg)@NPs, which deviate from this range. This behavior likely stems from two factors: (1) the substantial polymer quantities used during coating may incorporate more magnetite nanoparticles into single composite particles, and (2) high molecular weight polymers can significantly increase hydrodynamic diameter through thicker coating layers.

**Figure 2:**
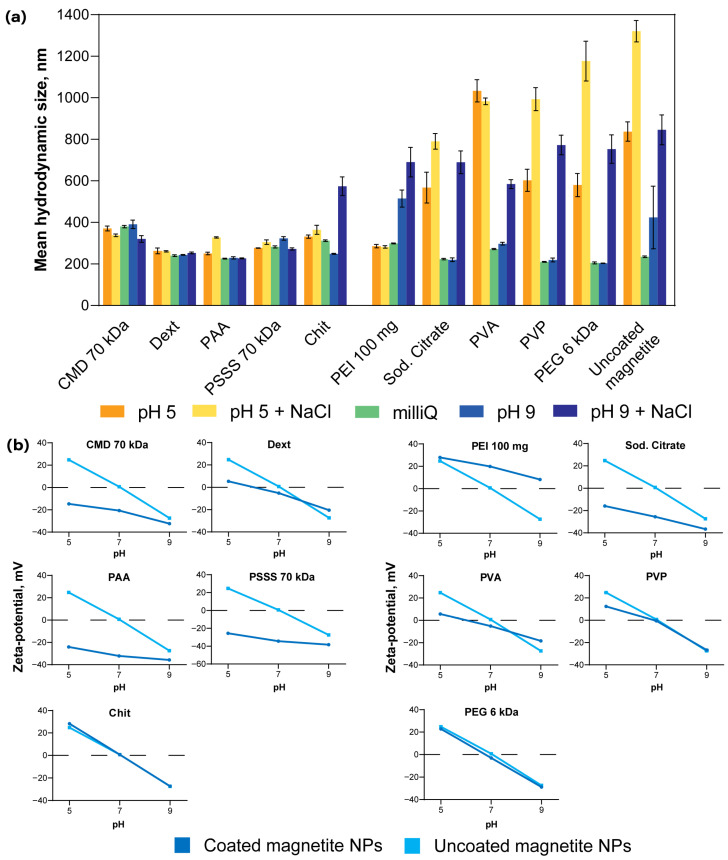
Characterization of coated and uncoated magnetite nanoparticles at different pH and in solutions with different ionic strengths. (**a**) Mean hydrodynamic size; (**b**) Zeta potential. Data are presented as means of individual measurements ± standard deviations, *n* = 3.

To verify the successful magnetite coating using our protocols, we measured the hydrodynamic diameter and zeta potential for both uncoated nanoparticles and particles coated with CMD 70 kDa, Dext, PAA, PSSS 70 kDa, Chit, PEI 100 mg, sodium citrate, PVA, PVP, and PEG 6 kDa at pH 5 and pH 9 both under low ionic strength (10 mM buffers) and high ionic strength conditions (150 mM NaCl) (Figure 2).

Uncoated magnetite exhibits behavior typical of poorly charge-stabilized particles: rapid coagulation occurs even in 10 mM buffers, with hydrodynamic diameters reaching ~1000 nm in 150 mM NaCl. In contrast, CMD 70 kDa, Dext, PAA, and PSSS 70 kDa coatings provide robust steric stabilization at both pH 5 and 9, as evidenced by unchanged hydrodynamic sizes. PEI and Chit demonstrate effective stabilization at pH 5, where both polymers maintain a strong positive charge (see zeta potential data), but fail to stabilize at pH 9, because this value is close to their pH_iep_. Sodium citrate does not provide steric stabilization, since citrate is a small ion that is suitable for this purpose only up to certain ionic strengths (~60–100 mM) [66], but successful coating is confirmed by negative zeta potential values across the studied pH range due to carboxyl groups.

The situation is less clear-cut for the three remaining coatings: PVA, PVP, and PEG 6 kDa. All of them fail to provide good steric stabilization, leaving just indirect evidence available for discussion. For PVA-coated nanoparticles, we observe stability at pH 9 and 10 mM ionic strength (certainly, it is a rather low value, yet sufficient to coagulate uncoated particles). Additionally, the slope of the zeta-pH dependence is significantly reduced compared to magnetite nanoparticles. We speculate these results from the polymer blocking protonatable/deprotonatable sites on the nanoparticle surface, thereby confirming successful coating. Moreover, previous studies have reported PVA coating using sufficiently similar protocols, where successful coating formation was unequivocally confirmed by Fourier transform infrared spectroscopy (FTIR) and/or thermogravimetric analysis (TGA) [67,68,69].

A similar pattern emerges for PVP-coated nanoparticles, though the identical zeta potential values of PVP@NPs and uncoated nanoparticles at pH 9 somewhat reduce confidence in coating efficacy. However, the available data, supported by literature reports of nanoparticle coating with PVP using similar methodologies [70,71] (with FTIR-confirmed coating), provide sufficient evidence to conclude successful PVP coating of the nanoparticles.

Finally, for PEG 6 kDa coating, we observed no differences from uncoated nanoparticles in either zeta potential or behavior under elevated ionic strength (150 mM). The sole detectable difference—a marginally reduced size in MilliQ water—was unequivocally insufficient to confirm successful coating. Consequently, PEG@NPs were excluded from further investigations.

The lectins, ConA and SBA, were covalently immobilized onto the surface of (CMD10–20 kDa)@NPs via carbodiimide chemistry. Successful conjugation was confirmed by lateral flow assay using test strips with immobilized glycogen and mucin at the test lines, respectively [72] (the test strips for ConA are shown in Figure S2).

Table S1 summarizes the coating protocols and the corresponding quality assessment. From the initial set of 18 coated nanoparticles, we proceeded with in vivo testing of 17 selected variants.

### 3.3. Magnetite Nanoparticle Behavior in Blood Serum

When studying the behavior of nanoparticles in biological systems, it is essential to consider their interactions with blood serum. This includes two key phenomena: potential nanoparticle aggregation due to the high ionic strength of biological fluids, and the adsorption of proteins and other biomolecules onto the nanoparticle surface, leading to the formation of the so-called protein or biomolecular corona [73,74].

We investigated the morphology of coated nanoparticles (specifically, with CMD 150 kDa, Chit, PEI 100 mg, and PSSS 500 kDa) in detail by TEM, both before and after incubation in blood serum. As shown in the obtained images (Figure S3), magnetite cores of approximately the same size as those in the uncoated sample (19 ± 4 nm, Figure 1b) were assembled into aggregates ranging from 200 to 500 nm in diameter, which is in good agreement with DLS data (Figure 1c). It is important to note, however, that standard TEM sample preparation involves drying, which may lead to the formation of such artifacts. Following serum incubation, no substantial changes were detected, with the possible exception of traces of a protein corona. However, proteins consist primarily of light atoms and scatter electrons weakly compared to nanoparticles, resulting in low contrast and poor visibility. Additionally, sample drying may also cause the collapse of the protein corona and alter its native morphology and thickness. Therefore, a more accurate assessment of the protein corona’s size could be achieved using cryo-TEM or the simpler DLS method.

In this work, to investigate the effect of various coatings on nanoparticle behavior in serum, their size and zeta potential were further analyzed using DLS. To ensure that protein aggregates and other particulates present in serum did not affect the measurements, the laser power was kept constant across all experiments, and the scattering intensity was recorded for each sample. The scattering intensity for pure serum was more than tenfold lower than for all nanoparticle-containing samples, indicating that its contribution to the overall signal was negligible (Figure S4).

**Figure 3:**
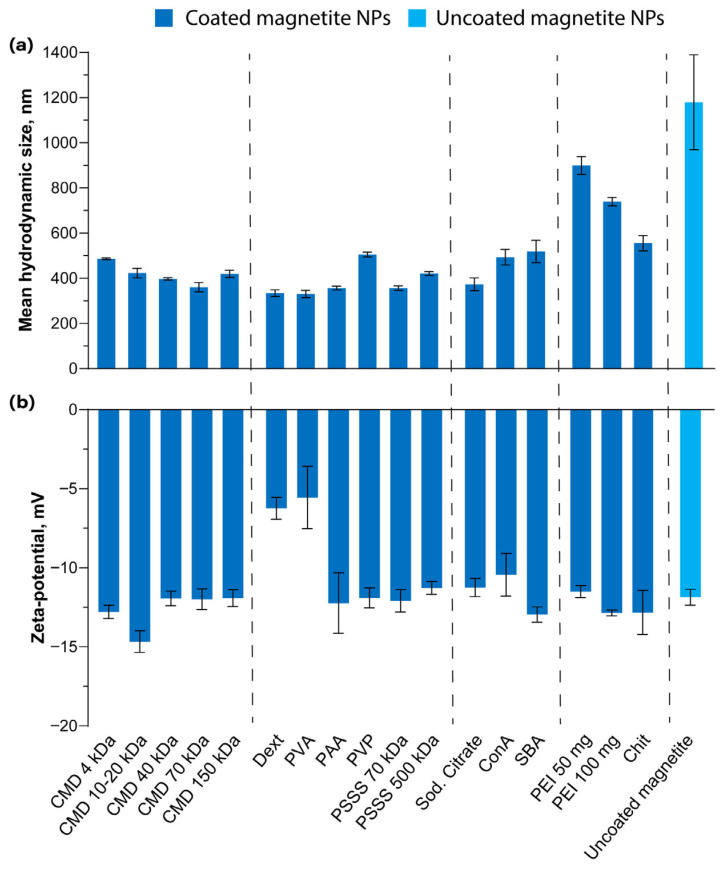
DLS analysis of nanoparticle behavior in blood serum: (**a**) mean hydrodynamic size and (**b**) zeta potential. Dashed lines are used to visually align the names of the coatings with their corresponding bars.

Despite the considerable variability in the zeta potential of nanoparticles measured in dilute buffer solutions, their values converge after even brief incubation with serum (Figure 3b). This aligns with the observation that most nanoparticles acquire a similar zeta potential in serum [75,76]. The only samples exhibiting a statistically significant difference from uncoated magnetite are those coated with PEI 100 mg (–12.9 mV, *p* = 0.03), CMD 10–20 kDa (–14.7 mV, *p* = 0.005), Dext (–6.2 mV, *p* = 0.0003), and PVA (–5.6 mV, *p* = 0.006). For PEI-coated NPs, this likely results from strong interactions between the highly cationic PEI and serum proteins [77], leading to extensive protein adsorption that fully compensates the initial surface charge and even induces marked charge reversal, as previously demonstrated [31]. For CMD 10–20 kDa, we attribute the observed difference to the highly effective coating and the high initial negative charge of the nanoparticles. However, given the relatively small absolute differences in zeta potential from uncoated particles and the limited number of measurements (*n* = 3), we consider these results to be of questionable significance. In the case of Dext and PVA, we speculate that the notably lower zeta potential magnitudes may be attributed to the intrinsically neutral nature of these polymers, resulting in reduced serum protein adsorption. However, this finding diverges from a prior comparative study of Dext- and CMD-coated particles, which reported nearly identical zeta potentials [78].

Regarding size (Figure 3a), uncoated particles exhibit a substantial increase, most likely attributable to coagulation, an effect also observed in buffer solutions. Following short-term incubation in serum, most coated nanoparticles maintained hydrodynamic radii comparable to their pre-incubation values. We propose that this stability is largely due to the initially relatively large hydrodynamic size of the nanoparticles used in this study, rendering the contribution of the adsorbed protein corona to the overall hydrodynamic diameter practically negligible. A notable size increase was primarily observed for PEI-coated nanoparticles, presumably resulting from the adsorption of a large number of proteins onto the cationic PEI, leading to the rapid formation of large aggregates. This finding is entirely consistent with previously reported data [79,80,81]. A certain degree of size increase was also observed for cationic Chit, which can be explained by the same mechanism.

Thus, our study presents size and zeta potential data for nanoparticles with an exceptionally wide variety of coatings, including after incubation in serum, offering particular interest in the context of existing literature. The obtained data are consistent with the current understanding in the field, with rare exceptions separately discussed above. Nevertheless, even these exceptions can be explained by the complex nature of protein corona formation. It has previously been demonstrated that not only surface charge and the type of adsorbed polymer, but even nanoparticle size can significantly influence the composition of the protein corona, with notable effects observed for size variations as small as a factor of two (from 50 to 100 nm) [82].

### 3.4. Measurement of Coated Magnetite Nanoparticle Circulation Kinetics and Circulation Half-Life in the Bloodstream of Mice

**Figure 4:**
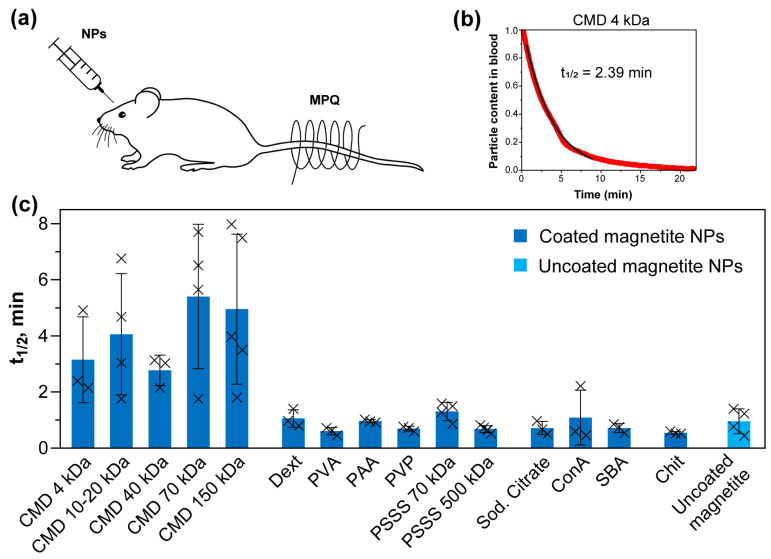
Circulation kinetics of coated and uncoated magnetite nanoparticles. (**a**) Schematic representation of the noninvasive in vivo measurement of NP kinetics using MPQ method; (**b**) Representative circulation kinetics of (CMD 4 kDa)@NPs (magnetic signal was normalized to the maximum). The red solid line shows the data over the entire signal range of the clearance curve. The black line shows the monoexponential fit that was used to calculate the circulating half-life in the 80% range; (**c**) Calculated circulation half-life for different nanoparticles. Crosses indicate individual values, *n* ≥ 3.

We next studied the circulation kinetics of coated NPs in the mouse bloodstream using the MPQ method (Figure 4). The tail of the anesthetized mouse was placed in the coil of the device, and then nanoparticles were injected intravenously into the retro-orbital sinus. The magnetic signal was recorded every 1.6 s until it completely dropped to the noise level. The method is a non-invasive alternative to the widely used method of periodic blood sampling to determine the content of nanomaterials in the bloodstream at various time points. The MPQ technique avoids the adverse effects of intervention in the animal’s body, which can significantly affect the kinetics of nanoparticles due to, for example, a decrease in total blood volume, changes in blood pressure, pulse rate, and other important parameters. There are other non-invasive methods for studying the kinetics of nanomaterials, such as dynamic MRI [83,84]. However, this technique is limited to detecting a relatively narrow concentration range and also involves relatively complex experimental procedures. The MPQ method, in turn, allows monitoring the kinetics in real time. In addition, the low noise level of the device, achieved by measuring the magnetic signal of only nonlinear magnetic materials, allows for the detection of nanoparticles with particularly high sensitivity [28].

Statistically significant differences compared to t_1/2_ of uncoated magnetite nanoparticles were calculated using a two-tailed Welch’s *t*-test. We revealed differences for CMD-coated NPs, in particular for (CMD 40 kDa)@NPs (**, *p* ≤ 0.01), (CMD 70 kDa)@NPs (*, *p* ≤ 0.05), and (CMD 150 kDa)@NPs (*, *p* ≤ 0.05), which is consistent with the results obtained previously [28] when comparing the circulation of fluidMAG magnetic iron oxide nanoparticles (Chemicell, Germany) with different coatings.

However, for other coatings, we observed no significant differences in circulation kinetics or half-life. A possible explanation for this observation is that while the coating type influences the composition of the protein corona formed on nanoparticles upon intravenous administration [82], the corona itself tends to give NPs a zeta potential in the range of −10 to −20 mV, regardless of the initial surface characteristics [85]. For instance, Alkilany et al. demonstrated that both anionic and cationic polyelectrolyte-coated gold nanorods, when incubated in biological media containing bovine serum albumin, acquired identical zeta potentials (−20 mV) [76]. These findings were also confirmed for most of the nanoparticles in the present study (Figure 3).

The blood circulation kinetics of PEI-coated nanoparticles have not been studied due to the low magnetic signal of the nanoparticles in the animal’s tail vein. Apparently, due to the cationic nature of this polymer, the NPs demonstrated strong binding with both plasma proteins (Figure 3) and blood cellular components, significantly impairing their circulatory performance [77].

### 3.5. Biodistribution and Tumor Accumulation of Coated Magnetite Nanoparticles

Next, we used the MPQ method to quantitatively analyze the accumulation of various coated and uncoated magnetite nanoparticles in the organs and tissues of tumor-bearing mice [86]. For this purpose, 2 × 10^6^ CT26 cells in 200 µL of culture medium were inoculated subcutaneously in the right flank of BALB/c female mice. When the tumor reached the size of 600 mm^3^, two experimental groups of mice were formed. The first group was retroorbitally injected with 1000 µg of NPs, then after 24 h, the mice were sacrificed, and the magnetic signal was measured in the following organs and tissues: heart, lungs, liver, spleen, kidneys, tumor, brain, bone, muscle, and blood. For the second group of mice, the experiment was carried out similarly, but with two changes: (1) the magnetic nanoparticles were focused in the tumor area by the magnetic field of the applied magnet, (2) the magnetic signal in the organs and tissues was measured after 3 h of nanoparticle circulation.

**Figure 5:**
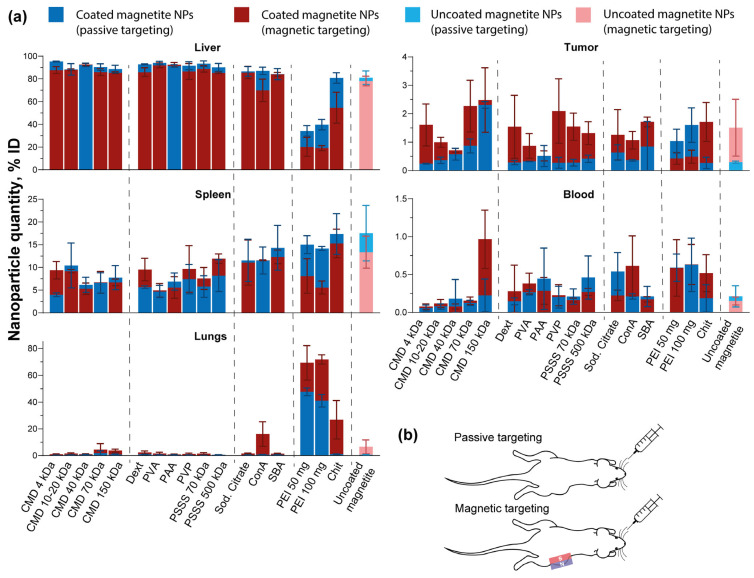
Biodistribution and tumor accumulation of coated and uncoated magnetite nanoparticles. (**a**) Accumulation of nanoparticles in some organs and tissues, *n* ≥ 3. Dashed lines are used to visually align the names of the coatings with their corresponding bars; (**b**) Scheme of implementation of passive and magnetic targeting of nanoparticles to the tumor. The magnet is represented by a red-and-blue rectangle.

This experimental setup allowed us to compare two types of solid tumor targeting—passive and magnetic (Figure 5b). The principle of passive targeting is that fast-growing tumors build a developed system of blood vessels to obtain nutrients from the body, and under conditions typical for tumors (such as inflammation, hypoxia, etc.), the vascular endothelium becomes more permeable than that of healthy tissues, providing increased penetration of nanoparticles to tumor cells [87]. With magnetic targeting, nanoparticles are additionally focused in the tumor area using an external magnetic field.

The results of the experiment are presented: (1) in Figure 5a for organs and tissues with delivery of more than 1% of the injected dose (liver, spleen, lungs, tumor) and blood; (2) in Figure S5 for delivery of less than 1% (kidneys, muscle, bone, brain, and heart). The accumulation of nanoparticles, expressed as both %ID/g of organ or tissue and %ID, is also presented in the form of heatmap tables (Table 2 and Table S2, respectively).

For most of the nanoparticle formulations tested, the biodistribution profiles were nearly identical and followed a characteristic pattern: approximately 80–90% of the injected dose accumulated in the liver, and 5–15% in the spleen, under both passive and magnetic targeting conditions.

The results obtained are consistent with the biodistribution profile of clinically approved iron oxide nanoparticles of comparable size—specifically, those not belonging to the ultrasmall superparamagnetic iron oxide class. For instance, nanoparticles coated with dextran (120–180 nm, Feridex I.V.) and carboxymethyldextran (60 nm, Resovist), used for MRI of hepatic tumors [88], are rapidly cleared from the bloodstream by cells of the mononuclear phagocyte system (MPS). This rapid clearance results in their accumulation in the liver and spleen, with approximately 80% and 6–10% of the injected dose, respectively [89]. While nanoparticles for gastrointestinal MRI (300–400 nm, GastroMARK) have also been approved [90], their oral administration makes a direct comparison of their biodistribution with that of intravenously administered particles irrelevant.

The high level of hepatic uptake is attributed to recognition and clearance of NPs by resident Kupffer cells. The process appears to be so rapid and efficient that applying a magnetic field over the tumor site had minimal impact on liver accumulation. Indeed, for the majority of nanoparticle types, no statistically significant differences were observed between magnetic and passive delivery. However, statistical analysis revealed significant differences in liver accumulation between magnetic and passive targeting for several formulations: (PEI 100 mg)@NPs (**, *p* ≤ 0.01), as well as (PEI 50 mg)@NPs, Chit@NPs, (CMD 4 kDa)@NPs, and Dext@NPs (*, *p* ≤ 0.05). In the case of PEI- and Chit-coated NPs, the observed effect of the magnetic field may be explained by their comparatively lower hepatic uptake. Due to the cationic nature of these polymers, the nanoparticles preferentially accumulated in the lungs—ranging from 40 to 50% under passive targeting (except for Chit@NPs) and 30–70% under magnetic targeting. This pattern likely results from interactions with both plasma proteins and blood cellular components [77].

ConA@NPs also showed high accumulation in the lungs when magnetic targeting was implemented—16.1 ± 9.2%, probably due to the specific interaction with carbohydrate molecules on the cell surface. With passive targeting, the percentage of accumulation in the lungs was significantly lower, possibly due to the fact that the signal was measured 24 h after administration, when all NPs had already redistributed from the lungs to the liver and spleen.

For nearly all nanoparticle types, magnetic targeting resulted in higher average tumor accumulation compared to passive targeting. Statistically significant differences between the two delivery methods were observed for CMD 4 kDa-, PVP-, PSSS 70 kDa-, and Chit-coated NPs (Figure S6). During passive targeting, statistically significant differences in tumor delivery efficiency compared to uncoated magnetite nanoparticles were observed only for (CMD 150 kDa)@NPs (***, *p* ≤ 0.001), likely attributable to their extended circulation in the bloodstream. In contrast, no significant differences were found for any of the coated nanoparticles compared to uncoated magnetite nanoparticles during magnetic targeting. Additionally, a strong positive correlation was observed (R = 0.972 according to a Pearson correlation analysis), indicating that CMD-coated nanoparticles with higher molecular weight chains exhibited increased tumor delivery efficiency under passive targeting conditions.

However, universal strategies exist to prolong nanoparticle circulation (and thereby enhance accumulation in target organs and tissues) that function independently of surface coating properties. These approaches primarily rely on direct modulation of the MPS. For instance, MPS suppression can be achieved by depleting macrophages with liposomal clodronate, by pre-administering various blocking agents (e.g., high doses of non-toxic nanoparticles) to temporarily saturate phagocytic activity [91,92,93], or through MPS-cytoblockade technology, which uses anti-erythrocyte antibodies to redirect MPS clearance toward red blood cells, thereby substantially extending nanocarrier circulation time [3,27]. Such interventions alter nanoparticle biodistribution—specifically reducing accumulation in the liver and other MPS organs (spleen, lungs, bone marrow) while increasing tumor uptake.

**Figure 6:**
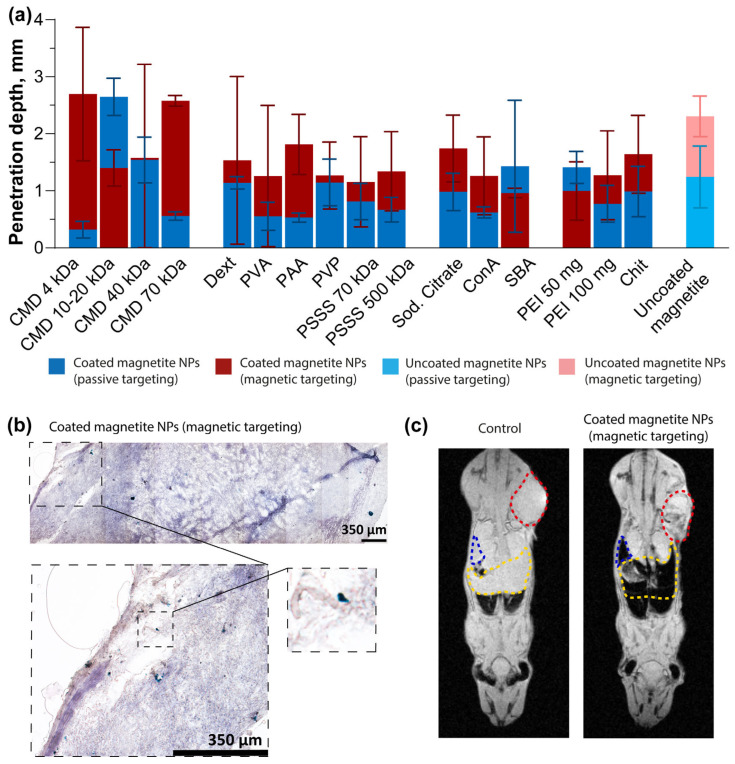
Histological analysis of tumors and biodistribution studied by MRI. (**a**) Penetration depth of nanoparticles into the tumors 24 h after administration of NPs during passive targeting and 3 h after administration of NPs during magnetic targeting, *n* ≥ 2; (**b**) Representative histological section of tissue from mice that were injected with Dext@NPs; (**c**) The biodistribution of (CMD 70 kDa)@NPs in mice studied with MRI, *n* = 3. Untreated mice served as the control. Livers, spleens, and tumors are highlighted with yellow, blue, and red dotted lines, respectively.

To validate the biodistribution data acquired with MPQ, we conducted a complementary investigation using MRI. The MRI results (Figure 6c) for (CMD 70 kDa)@NPs demonstrated a pronounced nanoparticle accumulation in the mononuclear phagocyte system organs, particularly the liver and spleen, while showing comparatively much lower uptake in tumor tissue. The obtained biodistribution profile confirms the data of the MPQ analysis.

### 3.6. Histological Analysis of Tumors

Next, we performed histological analysis of the extracted tumors to determine the penetration depth of nanoparticles with different coatings (Figure 6a). The tissues were cut into 7 µm-thick sections on a Cryotome FSE cryostat (Thermo Scientific, USA). Staining was performed with hematoxylin/eosin, as well as potassium hexacyanidoferrate(II) according to the Perls method, commonly used to determine the presence of iron in tissues. The samples were then examined on an Olympus Fluoview FV3000 confocal microscope (Olympus, Japan) in bright field mode (Figure 6b and Figure S7).

In passive targeting, coated nanoparticles showed no statistically significant differences in penetration depth compared to uncoated ones. However, under magnetic targeting, PVP@NPs, SBA@NPs, and (PEI 50 mg)@NPs exhibited significantly reduced penetration (Figure S8a). Notably, when comparing targeting methods, (CMD 4 kDa)@NPs, (CMD 70 kDa)@NPs, and PAA@NPs demonstrated deeper tissue penetration with magnetic targeting than with passive (Figure S8b). The maximum penetration depth for NPs delivered by passive targeting was 2.65 mm for (CMD 10–20 kDa)@NPs, while for NPs delivered by magnetic targeting, it was 2.69 mm for (CMD 4 kDa)@NPs.

## 4. Conclusions

In this work, we synthesized magnetite nanoparticles and modified them with 17 distinct coating types based on polymers, lectins, and small molecules. These coatings exhibited diverse physicochemical properties, including variations in molecular weight, charge, and functional group composition, resulting in nanoparticles with different sizes and zeta potentials. We then evaluated the nanoparticles’ in vivo performance by analyzing their blood circulation kinetics, biodistribution, tumor accumulation efficiency and penetration depth in mice. The study compared both passive and magnetic targeting approaches to assess their delivery efficacy.

Analysis of circulation kinetics revealed that among the tested coatings, only CMD-coated NPs with long polymer chains exhibited prolonged blood circulation time compared to uncoated magnetite nanoparticles. Biodistribution studies demonstrated enhanced passive tumor delivery efficiency for (CMD 150 kDa)@NPs, likely attributable to their extended circulation in the bloodstream. Furthermore, magnetic targeting improved tumor accumulation for certain nanoparticles compared to passive delivery. While clear trends were observed, the data indicate that polymer coatings—particularly their surface charge—significantly influence the accumulation profile of magnetite nanoparticles in the liver and lungs, but generally have minimal impact on tumor delivery efficiency. Additionally, magnetic field application not only enhanced tumor targeting for some nanoparticles but also increased their penetration depth into tumor tissue.

Our findings may contribute to the development of MPS inhibition strategies based on nanoparticle combinations. We propose that employing a mixture of nanoparticles with distinct organ biodistribution profiles could achieve more effective MPS blockade at a lower total dose, thereby leading to a more substantial increase in tumor accumulation.

However, it is crucial to recognize that additional parameters—including nanoparticle size [94], magnetite synthesis methodology [34], administered dose [95], surface conjugation with targeting ligands [96], and other aspects such as various magnetic field parameters [97]—significantly impact nanoparticle kinetics and biodistribution profiles. Thus, the process of creating nanoagents that are effective for specific biological tasks may run into a long process of optimizing their composition.

The comprehensive investigation conducted in this work systematically demonstrates how surface coatings govern nanoparticle behavior in complex biological systems. The acquired data provide a robust foundation for rational design of nanoscale diagnostic and therapeutic agents, potentially streamlining their development pipeline and facilitating clinical translation.

## Figures and Tables

**Table 1:** Composition of coated magnetite nanoparticles.

No	Abbreviation	The Amounts of Nanoparticles and Coatings Used in the Modification
1	CMD 4 kDa	12 mg magnetite + 100 mg carboxymethyl dextran (4 kDa)
2	CMD 10–20 kDa	12 mg magnetite + 100 mg carboxymethyl dextran (10–20 kDa)
3	CMD 40 kDa	12 mg magnetite + 100 mg carboxymethyl dextran (40 kDa)
4	CMD 70 kDa	12 mg magnetite + 100 mg carboxymethyl dextran (70 kDa)
5	CMD 150 kDa	12 mg magnetite + 100 mg carboxymethyl dextran (150 kDa)
6	PEG 6 kDa	12 mg magnetite + 0.3 mg polyethylene glycol (6 kDa)
7	Dext	12 mg magnetite + 50 mg dextran (70 kDa)
8	PVA	12 mg magnetite + 9 mg polyvinyl alcohol (13–23 kDa)
9	PAA	12 mg magnetite + 100 mg polyacrylic acid (70 kDa)
10	PVP	12 mg magnetite + 100 mg polyvinylpyrrolidone (8 kDa)
11	PSSS 70 kDa	12 mg magnetite + 100 mg sodium polystyrene sulfonate (70 kDa)
12	PSSS 500 kDa	12 mg magnetite + 50 mg sodium polystyrene sulfonate (500 kDa)
13	Sod. Citrate	12 mg magnetite + 100 mg sodium citrate
14	ConA	1 mg CMD 10–20 kDa-coated magnetite + 0.1 mg concanavalin A (104–112 kDa)
15	SBA	1 mg CMD 10–20 kDa-coated magnetite + 0.1 mg soybean agglutinin (120 kDa)
16	PEI 50 mg	12 mg magnetite + 50 mg polyethylenimine (25 kDa)
17	PEI 100 mg	12 mg magnetite + 100 mg polyethylenimine (25 kDa)
18	Chit	12 mg magnetite + 7.5 mg chitosan (5 kDa)

**Table 2:** The accumulation of nanoparticles (%ID/g of organ or tissue) in the form of heatmap table for passive (P) and magnetic (M) targeting. Cells are color-coded on a red-yellow-green scale, where red indicates the minimum value and green indicates the maximum value.

	Targeting	Liver	Spleen	Lungs	Kidney	Kidney	Heart	Muscle	Bone	Brain	Blood	Tumor
**CMD 4 kDa**	P	71.25	14.88	1.40	0.35	0.27	0.05	0.06	0.17	0.02	0.04	0.12
	M	66.11	44.38	6.48	0.90	0.45	0.09	0.19	0.18	0.03	0.05	1.81
**CMD 10-20 kDa**	P	73.65	35.13	2.82	0.34	0.19	0.07	0.69	0.57	0.04	0.05	0.30
	M	62.91	32.63	4.57	0.44	0.30	0.09	0.13	0.12	0.02	0.17	1.25
**CMD 40 kDa**	P	56.72	20.48	3.46	0.20	0.46	0.17	0.27	1.44	0.02	0.12	0.27
	M	65.33	20.81	6.67	0.32	0.36	0.08	0.09	0.17	0.02	0.05	0.69
**CMD 70 kDa**	P	66.21	26.35	6.48	0.30	0.28	0.16	0.70	2.46	0.06	0.08	0.42
	M	62.19	24.73	27.88	0.72	0.73	0.47	0.76	0.90	0.05	0.05	1.91
**CMD 150 kDa**	P	53.97	27.34	4.80	0.21	0.25	0.10	0.25	2.52	0.04	0.15	0.79
	M	64.44	23.31	21.38	0.74	0.76	0.28	0.88	0.89	0.05	0.64	1.16
**Dext**	P	73.97	31.49	5.11	0.22	0.21	0.08	0.39	0.20	0.10	0.10	0.26
	M	58.77	52.02	14.45	0.44	0.72	0.17	0.19	0.37	0.09	0.19	1.59
**PVA**	P	59.01	21.27	2.65	0.19	0.34	0.18	0.16	0.34	0.15	0.18	0.30
	M	63.07	27.33	8.25	0.33	0.37	0.27	0.19	0.39	0.13	0.25	1.06
**PAA**	P	60.41	35.99	1.69	0.48	0.48	0.28	0.36	0.65	0.25	0.30	0.52
	M	64.21	23.25	4.49	0.44	0.39	0.19	0.21	0.46	0.11	0.19	0.58
**PVP**	P	61.43	34.05	1.61	0.34	0.19	0.11	0.20	0.16	0.16	0.13	0.19
	M	56.37	48.77	5.95	0.60	0.32	0.16	0.32	0.27	0.07	0.15	2.32
**PSSS 70 kDa**	P	58.70	33.59	0.85	0.25	0.14	0.29	0.12	0.30	0.15	0.14	0.26
	M	61.85	39.20	8.49	0.49	0.35	0.21	0.83	0.58	0.11	0.11	1.59
**PSSS 500 kDa**	P	59.21	40.72	1.96	0.21	0.41	0.35	0.35	0.86	0.18	0.38	0.35
	M	61.50	63.33	3.19	0.59	0.57	0.38	0.59	0.99	0.23	0.18	1.31
**Sod. Citrate**	P	58.53	59.70	1.73	0.43	0.54	0.32	0.40	0.37	0.33	0.36	0.60
	M	61.62	63.24	9.66	0.60	0.45	0.43	0.39	0.42	0.12	0.15	1.38
**ConA**	P	58.37	47.38	3.06	0.27	0.25	0.14	0.12	0.45	0.29	0.14	0.36
	M	51.70	54.55	103.80	1.39	1.36	0.72	0.33	0.69	0.14	0.41	1.40
**SBA**	P	51.36	73.68	24.75	0.91	1.12	0.62	0.95	1.20	0.61	0.89	0.85
	M	54.53	57.29	8.39	0.58	0.36	0.35	0.30	0.80	0.13	0.12	1.67
**PEI 50 mg**	P	23.30	76.97	261.21	2.42	2.22	4.47	0.29	2.09	0.16	0.39	0.96
	M	15.16	38.25	368.39	2.98	2.73	4.65	0.31	0.34	0.16	0.39	0.45
**PEI 100 mg**	P	29.08	79.55	202.44	3.23	3.53	7.20	0.65	2.24	0.69	0.76	1.15
	M	14.32	26.36	422.44	4.73	5.02	7.44	0.44	0.53	0.50	0.42	0.46
**Chit**	P	45.79	73.19	5.35	0.39	0.18	0.12	0.18	0.27	0.31	0.12	0.17
	M	42.36	88.79	115.32	1.62	1.49	0.57	0.23	0.52	0.24	0.31	2.10
**Uncoated magnetite**	P	55.95	92.93	3.59	0.78	0.39	0.34	0.32	0.36	0.07	0.14	0.27
	M	44.57	67.83	39.29	0.55	0.48	0.21	0.25	0.33	0.07	0.10	1.31

## Data Availability

The original contributions presented in this study are included in the article/Supplementary Materials. Further inquiries can be directed to the corresponding author(s).

## Supplementary Materials

The following supporting information can be downloaded at: https://www.mdpi.com/article/10.3390/pharmaceutics18030345/s1, Supplementary Note S1: Magnetic field induction of the permanent magnet employed for the focusing of nanoparticles in the tumor area; Figure S1: Magnetic field induction of the permanent magnet as a function of the distance from the magnet surface; Figure S2: Analysis of concanavalin A-coated nanoparticles by lateral flow assay; Figure S3: TEM images of coated magnetite nanoparticles before and after incubation in blood serum; Figure S4: Scattering intensity for pure serum and nanoparticle samples in serum; Figure S5: Accumulation in some organs and tissues of less than 1% of coated and uncoated magnetite nanoparticles; Figure S6: Tumor accumulation of coated and uncoated magnetite nanoparticles; Figure S7: Representative histological sections of tissues from mice injected with different nanoparticles under various targeting conditions; Figure S8: Statistically significant difference in tumor tissue penetration depth (a) between coated and uncoated magnetite nanoparticles with magnetic targeting; (b) between passive and magnetic targeting for coated and uncoated magnetite nanoparticles. Table S1: Summary of coating protocols and coating quality assessment for the 18 formulations; Table S2: The accumulation of nanoparticles (%ID) in the form of a heatmap table.

## Abbreviations

The following abbreviations are used in this manuscript:

MPQ	Magnetic particle quantification
SPIONs	Superparamagnetic iron oxide nanoparticles
MRI	Magnetic resonance imaging
FDA	Food and Drug Administration
PEG	Polyethylene glycol
SDS	Sodium dodecyl sulfate
NPs	Nanoparticles
SEM	Scanning electron microscopy
TEM	Transmission electron microscopy
CMD	Carboxymethyldextran
Chit	Chitosan
Dext	Dextran
PVA	Polyvinyl alcohol
PAA	Polyacrylic acid
PVP	Polyvinylpyrrolidone
PSSS	Sodium polystyrene sulfonate
SBA	Soybean agglutinin
ConA	Concanavalin A
DLS	Dynamic light scattering
FTIR	Fourier transform infrared spectroscopy
TGA	Thermogravimetric analysis
PEI	Polyethyleneimine
