# Supplementary material for: Effect of Different Magnetite Nanoparticle Coatings on Blood Circulation, Biodistribution, Tumor Accumulation and Penetration
Source: Pharmaceutics. 2026 Mar 11;18(3):345. doi: 10.3390/pharmaceutics18030345 (PMC13029123; doi:10.3390/pharmaceutics18030345)
Supplement: Supplementary file 1 [file pharmaceutics-18-00345-s001.zip › pharmaceutics-4129259-supplementary.pdf]

Supplementary materials

Effect of different magnetite nanoparticle coatings on blood circulation, biodistribution, tumor accumulation and penetration

Elizaveta N. Mochalova, Maria A. Yurchenko, Tatiana S. Vorobeva, Darina A. Maedi, Nikita O. Chernov, Olga A. Kolesnikova, Ekaterina D. Tereshina, Victoria O. Shipunova, Maria N. Yakovtseva, Petr I. Nikitin, Maxim P. Nikitin***

* mochalova.en@talantiuspeh.ru

** nikitin.mp@talantiuspeh.ru

Table S1. Summary of coating protocols and coating quality assessment for the 18 formulations; * – due to the absence of verifiable coating on the nanoparticles, these formulations were excluded from the study.

**№**	**Abbre viation**	**Coating protocol**	**Coating quality verification**
1	CMD 4 kDa	12 mg of magnetite NPs in 100 µl of water were mixed with 100 mg of CMD 4 kDa sodium salt (Carbosynth Ltd., YC64857) in 300 µl of MilliQ water. The resulting suspension was placed in an ultrasonic bath for a few seconds and then incubated in a water bath at 90 °C for 5 minutes. After that, the NPs were cooled at 4 °C for another 5 minutes. This procedure was repeated three times, and the mixture was left to incubate overnight at RT. The coated NPs were centrifuged for 10 min at 10,000 g, and then washed three times with 2 ml of MilliQ water by centrifugation in order to remove unbound polymer.	Highly negative zeta potential by DLS
2	CMD 10-20 kDa	12 mg of magnetite NPs in 100 µl of water were mixed with 100 mg of CM-Dextran sodium salt BioXtra (Sigma-Aldrich, 86524-50G-F) in 300 µl of MilliQ water. The resulting suspension was placed in an ultrasonic bath for a few seconds and then incubated in a water bath at 90 °C for 5 minutes. After that, the NPs were cooled at 4 °C for another 5 minutes. This procedure was repeated three times, and the mixture was left to incubate overnight at RT. The coated NPs were centrifuged for 10 min at 10,000 g, and then washed three times with 2 ml of MilliQ water by centrifugation in order to remove unbound polymer.	Highly negative zeta potential by DLS
3	CMD 40 kDa	12 mg of magnetite NPs in 100 µl of water were mixed with 100 mg of CMD 40 kDa sodium salt (Carbosynth Ltd., YC64859) in 300 µl of MilliQ water. The resulting suspension was placed in an ultrasonic bath for a few seconds and then incubated in a water bath at 90 °C for 5 minutes. After that, the NPs were cooled at 4 °C for another 5 minutes. This procedure was repeated three times, and the mixture was left to incubate overnight at RT. The coated NPs were centrifuged for 10 min at 10,000 g, and then washed three times with 2 ml of MilliQ water by centrifugation in order to remove unbound polymer.	Highly negative zeta potential by DLS

4	CMD 70 kDa	12 mg of magnetite NPs in 100 µl of water were mixed with 100 mg of CMD 70 kDa sodium salt (Carbosynth Ltd., YC64860) in 300 µl of MilliQ water. The resulting suspension was placed in an ultrasonic bath for a few seconds and then incubated in a water bath at 90 °C for 5 minutes. After that, the NPs were cooled at 4 °C for another 5 minutes. This procedure was repeated three times, and the mixture was left to incubate overnight at RT. The coated NPs were centrifuged for 10 min at 10,000 g, and then washed three times with 2 ml of MilliQ water by centrifugation in order to remove unbound polymer.	Highly negative zeta potential and excellent colloidal stability in 0.15 M NaCl by DLS
5	CMD 150 kDa	12 mg of magnetite NPs in 100 µl of water were mixed with 100 mg of CMD 150 kDa sodium salt (Carbosynth Ltd., YC64861) in 300 µl of MilliQ water. The resulting suspension was placed in an ultrasonic bath for a few seconds and then incubated in a water bath at 90 °C for 5 minutes. After that, the NPs were cooled at 4 °C for another 5 minutes. This procedure was repeated three times, and the mixture was left to incubate overnight at RT. The coated NPs were centrifuged for 10 min at 10,000 g, and then washed three times with 2 ml of MilliQ water by centrifugation in order to remove unbound polymer.	Highly negative zeta potential by DLS
6	PEG 6 kDa*	12 mg of magnetite NPs in 100 µl of water were mixed with 0.3 mg of polyethyleneglycole 6 kDa (Fluka Analytical, 03394-1KG) in 300 µl of MilliQ water. The resulting suspension was placed in an ultrasonic bath for a few seconds and then incubated in a water bath at 90 °C for 5 minutes. After that, the NPs were cooled at 4 °C for another 5 minutes. This procedure was repeated three times, and the mixture was left to incubate overnight at RT. The coated NPs were centrifuged for 10 min at 10,000 g, and then washed three times with 2 ml of MilliQ water by centrifugation in order to remove unbound polymer.	No significant differences from uncoated NPs by DLS
7	Dext	12 mg of magnetite NPs in 100 µl of water were mixed with 50 mg of dextran from Leuconostoc spp. 70 kDa (Sigma Aldrich, 31390-100G) in 300 µl of MilliQ water. The resulting suspension was placed in an ultrasonic bath for a few seconds and then incubated in a water bath at 90 °C for 5 minutes. After that, the NPs were cooled at 4 °C for another 5 minutes. This procedure was repeated three times, and the mixture was left to incubate overnight at RT. The coated NPs were centrifuged for 10 min at 10,000 g, and then washed three times with 2 ml of MilliQ water by centrifugation in order to remove unbound polymer.	Excellent colloidal stability in 0.15 M NaCl by DLS
8	PVA	12 mg of magnetite NPs in 100 µl of water were mixed with 9 mg of polyvinyl alcohol 13-23 kDa (Sigma-Aldrich, 363170-25G) in 300 µl of MilliQ water. The resulting suspension was placed in an ultrasonic bath for a few seconds and then incubated in a water bath at 90 °C for 5 minutes. After that, the NPs were cooled at 4 °C for another 5 minutes. This procedure was repeated three times, and the mixture was left to incubate overnight at RT. The coated NPs were centrifuged for 10 min at 10,000 g, and then washed three times with 2 ml of MilliQ water by centrifugation in order to remove unbound polymer.	The zeta potential-pH dependence differs from that of uncoated NPs
9	PAA	12 mg of magnetite NPs in 100 µl of water were mixed with 100 mg of polyacrylic acid (Sigma Aldrich, 323667-5G) in 300 µl of MilliQ water. The resulting suspension was placed in an ultrasonic bath for a few seconds and then incubated in a water bath at 90 °C for 5 minutes. After that, the NPs were cooled at 4 °C for another 5 minutes. This procedure was repeated three times, and the mixture was left to incubate overnight at RT. The coated NPs were centrifuged for 10 min at	Excellent colloidal stability in 0.15 M NaCl by DLS

		10,000 g, and then washed three times with 2 ml of MilliQ water by centrifugation in order to remove unbound polymer.	
10	PVP	12 mg of magnetite NPs in 100 µl of water were mixed with 100 mg of polyvinylpyrrolidone (Alfa Aesar, #41626) in 300 µl of MilliQ water. The resulting suspension was placed in an ultrasonic bath for a few seconds and then incubated in a water bath at 90 °C for 5 minutes. After that, the NPs were cooled at 4 °C for another 5 minutes. This procedure was repeated three times, and the mixture was left to incubate overnight at RT. The coated NPs were centrifuged for 10 min at 10,000 g, and then washed three times with 2 ml of MilliQ water by centrifugation in order to remove unbound polymer.	The zeta potential-pH dependence differs from that of uncoated NPs
11	PSSS 70 kDa	12 mg of magnetite NPs in 100 µl of water were mixed with 100 mg of poly(styrene sulfonic acid) sodium salt 70 kDa (Alfa Aesar, #41688) in 300 µl of MilliQ water. The resulting suspension was placed in an ultrasonic bath for a few seconds and then incubated in a water bath at 90 °C for 5 minutes. After that, the NPs were cooled at 4 °C for another 5 minutes. This procedure was repeated three times, and the mixture was left to incubate overnight at RT. The coated NPs were centrifuged for 10 min at 10,000 g, and then washed three times with 2 ml of MilliQ water by centrifugation in order to remove unbound polymer.	Highly negative zeta potential and excellent colloidal stability in 0.15 M NaCl by DLS
12	PSSS 500 kDa	12 mg of magnetite NPs in 100 µl of water were mixed with 100 mg of poly(styrene sulfonic acid) sodium salt 500 kDa (Alfa Aesar, #45050) in 300 µl of MilliQ water. The resulting suspension was placed in an ultrasonic bath for a few seconds and then incubated in a water bath at 90 °C for 5 minutes. After that, the NPs were cooled at 4 °C for another 5 minutes. This procedure was repeated three times, and the mixture was left to incubate overnight at RT. The coated NPs were centrifuged for 10 min at 10,000 g, and then washed three times with 2 ml of MilliQ water by centrifugation in order to remove unbound polymer.	Highly negative zeta potential by DLS
13	Sod. Citrate	12 mg of magnetite NPs in 100 µl of water were mixed with 100 mg of tri-sodium citrate 2-hydrate (Panreac Applichem, 131655.1210) in 300 µl of MilliQ water. The resulting suspension was placed in an ultrasonic bath for a few seconds and then incubated in a water bath at 90 °C for 5 minutes. After that, the NPs were cooled at 4 °C for another 5 minutes. This procedure was repeated three times, and the mixture was left to incubate overnight at RT. The coated NPs were centrifuged for 10 min at 10,000 g, and then washed three times with 2 ml of MilliQ water by centrifugation in order to remove unbound polymer.	Highly negative zeta potential by DLS
14	ConA	1 mg of (CMD10-20 kDa)@NPs in 15 µl of water was mixed with 85 µl of 0.1 M MES buffer (pH 5.0), containing 2 mg of EDC and 1 mg of sulfo-NHS. The resulting mixture was incubated for 20 min at RT. Then, the NPs were washed from unbound crosslinkers by centrifugation for 10 min at 10,000 g. After that, 200 µl of a Concanavalin A (Sigma-Aldrich, L7647-25MG) solution at a concentration of 0.5 g/l in 0.1 M HEPES buffer (pH 6.0) was added to the NPs and sonicated for 5 seconds. Finally, the NPs were incubated at 4-10 °C overnight and washed three times from unbound lectins by centrifugation.	Specific binding to the test line in the lateral flow assay
15	SBA	1 mg of (CMD10-20 kDa)@NPs in 15 µl of water was mixed with 85 µl of 0.1 M MES buffer (pH 5.0), containing 2 mg of EDC and 1 mg of sulfo-NHS. The resulting mixture was incubated for 20 min at RT. Then, the NPs were washed from unbound crosslinkers by centrifugation for 10 min at 10,000 g. After that, 200 µl of a soybean agglutinin (Vector Laboratories, USA) solution at a	Specific binding to the test line in the

		concentration of 0.5 g/l in 0.1 M HEPES buffer (pH 6.0) was added to the NPs and sonicated for 5 seconds. Finally, the NPs were incubated at 4-10 °C overnight and washed three times from unbound lectins by centrifugation.	lateral flow assay
16	PEI 50 mg	12 mg of magnetite NPs in 100 µl of water were mixed with 50 mg of branched polyethylenimine (Sigma-Aldrich, 408727-100ML) in 300 µl of MilliQ water. The resulting suspension was placed in an ultrasonic bath for a few seconds and then incubated in a water bath at 90 °C for 5 minutes. After that, the NPs were cooled at 4 °C for another 5 minutes. This procedure was repeated three times, and the mixture was left to incubate overnight at RT. The coated NPs were centrifuged for 10 min at 10,000 g, and then washed three times with 2 ml of MilliQ water by centrifugation in order to remove unbound polymer.	Positive zeta potential by DLS
17	PEI 100 mg	12 mg of magnetite NPs in 100 µl of water were mixed with 100 mg of branched polyethylenimine (Sigma-Aldrich, 408727-100ML) in 300 µl of MilliQ water. The resulting suspension was placed in an ultrasonic bath for a few seconds and then incubated in a water bath at 90 °C for 5 minutes. After that, the NPs were cooled at 4 °C for another 5 minutes. This procedure was repeated three times, and the mixture was left to incubate overnight at RT. The coated NPs were centrifuged for 10 min at 10,000 g, and then washed three times with 2 ml of MilliQ water by centrifugation in order to remove unbound polymer.	Highly positive zeta potential and excellent colloidal stability in 0.15 M NaCl (pH = 5) by DLS
18	Chit	12 mg of magnetite NPs in 100 µl of water were mixed with 7.5 mg of chitosan oligosaccharide lactate, 5 kDa (Sigma-Aldrich, 523682-1G) in 300 µl of MilliQ water. The resulting suspension was placed in an ultrasonic bath for a few seconds and then incubated in a water bath at 90 °C for 5 minutes. After that, the NPs were cooled at 4 °C for another 5 minutes. This procedure was repeated three times, and the mixture was left to incubate overnight at RT. The coated NPs were centrifuged for 10 min at 10,000 g, and then washed three times with 2 ml of MilliQ water by centrifugation in order to remove unbound polymer.	Excellent colloidal stability in 0.15 M NaCl (pH = 5) by DLS

Supplementary Note S1: Magnetic field induction of the permanent magnet employed for the focusing of nanoparticles in the tumor area.

We quantified the magnetic field of the permanent magnet employed for the focusing of nanoparticles in the tumor area using a Hall-effect-based magnetic induction meter РШ1-10 (Izmeritel, Russia). The dependence of the parameter on the distance from the magnet surface was obtained (Figure S1).

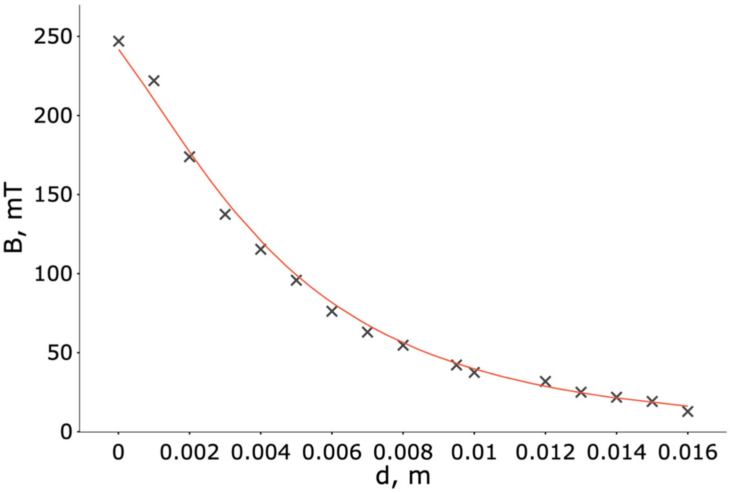

Figure S1. Magnetic field induction of the permanent magnet as a function of the distance from the magnet surface.

The experimental data show good agreement with the analytical fit obtained from the equation for the magnetic field along the axis of a rectangular magnet [Furlani, E.P. Permanent magnet and electromechanical devices: Materials, analysis, and applications; Academic: San Diego Calif., 2001, ISBN 0122699513].

$$B(z) = \frac{B_r}{\pi}\left(arctan\frac{2\cdot(z+L)\cdot\sqrt{a^2+b^2+4\cdot(z+L)^2}}{a\cdot b} - arctan\frac{2\cdot z\cdot\sqrt{a^2+b^2+4z^2}}{a\cdot b}\right)$$

where a and b are the length and width of the magnet, L is its height, z is the distance from the magnet surface to the measurement point, and Br is the remanence of the magnet. In our case, the fixed parameters were a = 0.02 m, b = 0.01 m, L = 0.005 m, yielding Br = 860 mT.

Given that the magnet was placed directly against the mouse tumor, and considering the average tumor depth of approximately 10 mm, the magnetic field within the tumor ranged from 250 mT to 50 mT. Therefore, to study the biodistribution and penetration depth of the nanoparticles, different regions of the tumor sample were analyzed.

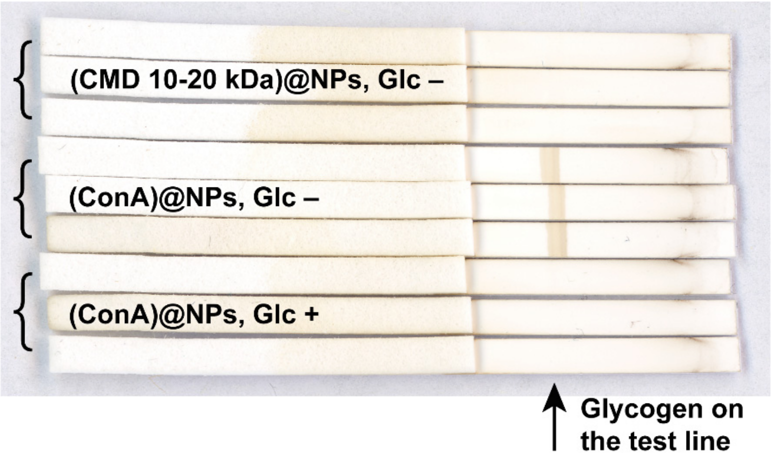

Figure S2. Analysis of concanavalin A-coated nanoparticles by lateral flow assay. ConA@NPs or control (CMD10-20 kDa)@NPs (1 μg) were suspended in 20 μL of 1% BSA in PBS, either in the presence (+) or absence (–) of 100 mM glucose (Glc). Test strips with glycogen on the test line (1 μL at 50 g/l concentration per 1 cm) were then placed in the suspensions to facilitate capillary migration. While no binding to the test line was observed for the control nanoparticles, ConA@NPs demonstrated clear interaction with the immobilized glycogen, thereby confirming effective lectin conjugation. The specificity of this binding was validated by its complete inhibition in glucose-supplemented buffer, indicating competitive binding to the free sugar. n = 3 for each test strip type.

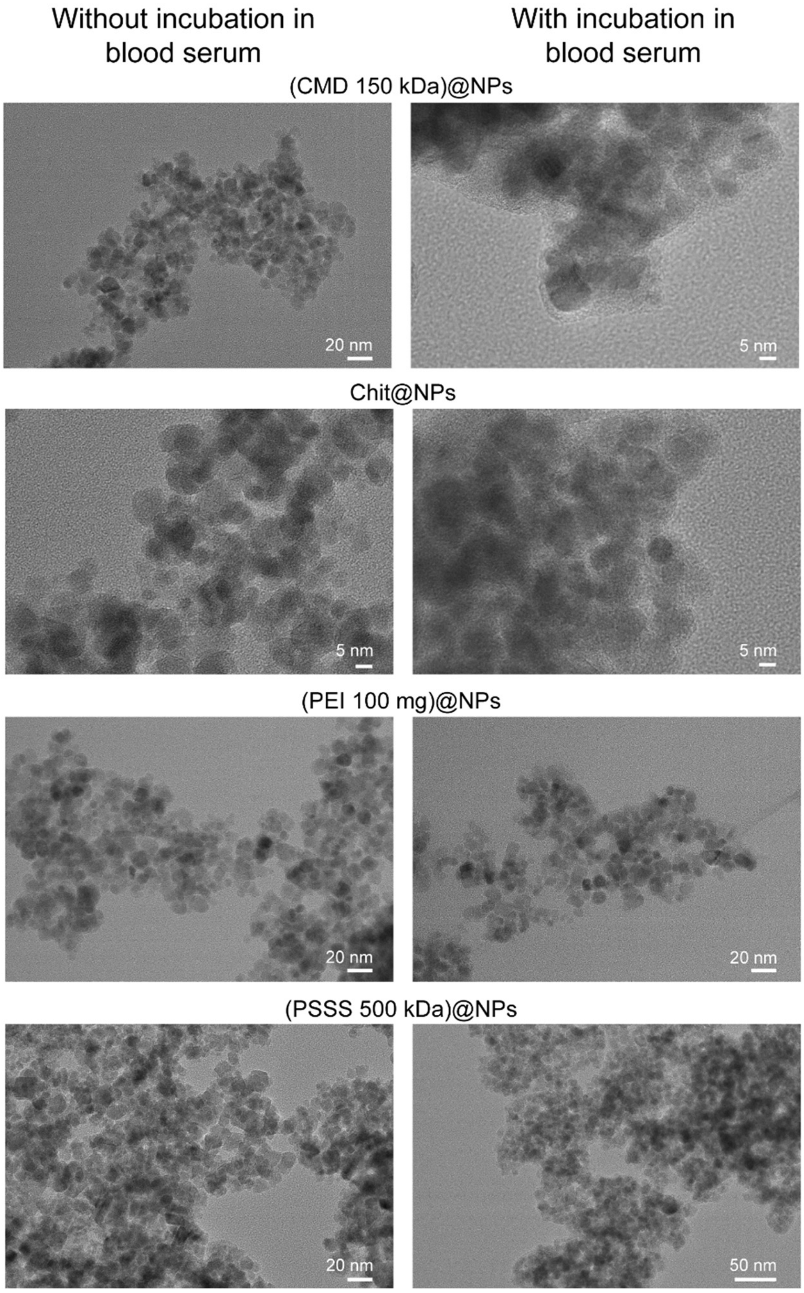

Figure S3. TEM images of coated magnetite nanoparticles before and after incubation in blood serum.

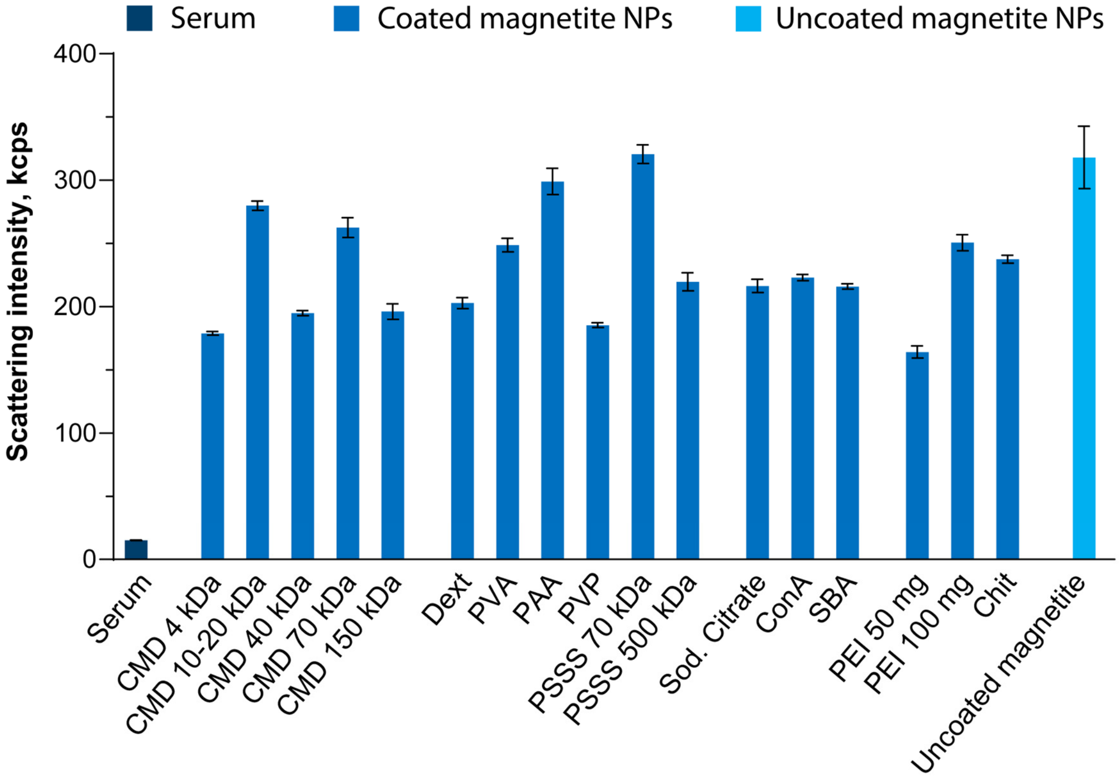

Figure S4. Scattering intensity for pure serum and nanoparticle samples in serum.

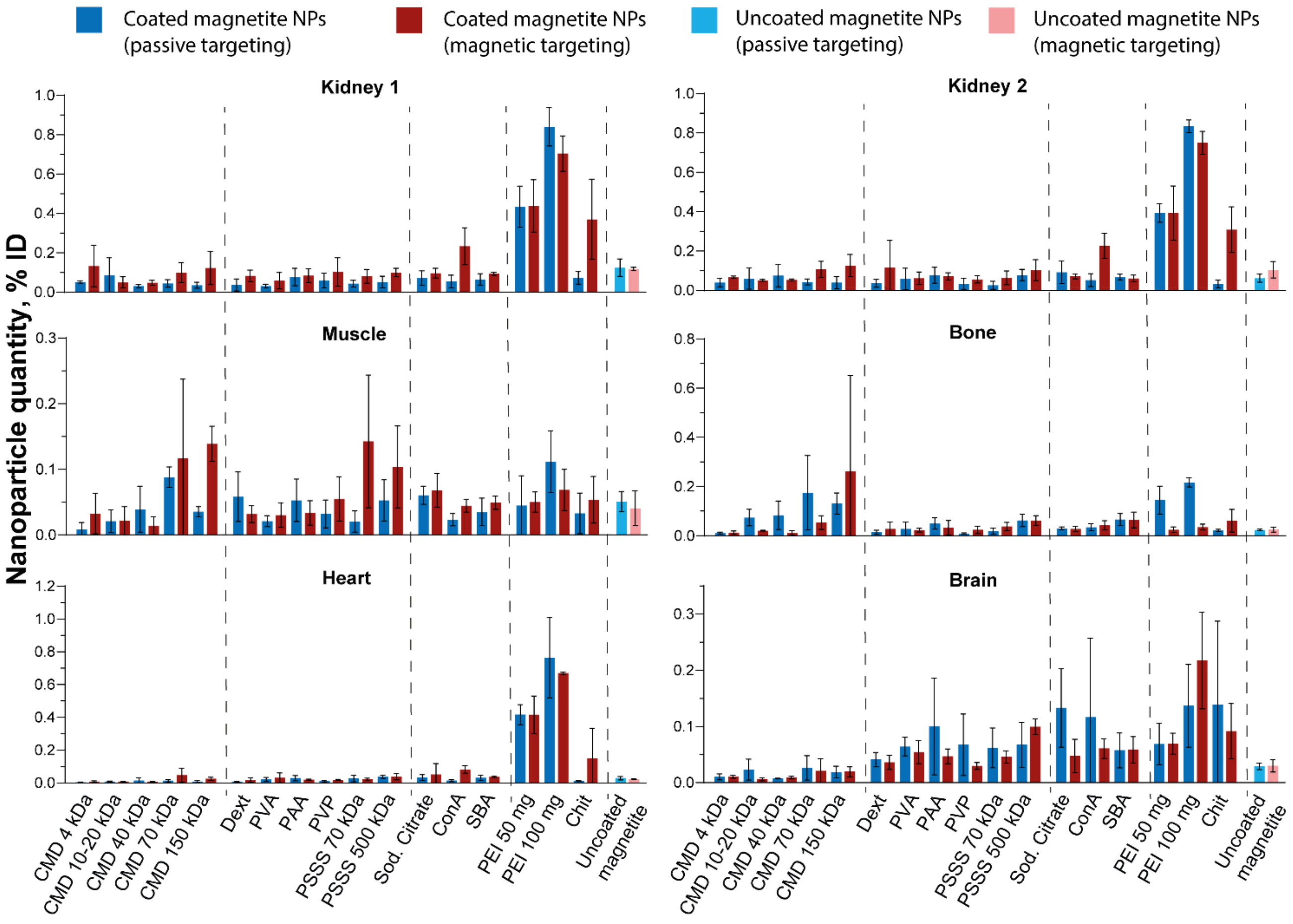

Figure S5. Accumulation in some organs and tissues of less than 1% of coated and uncoated magnetite nanoparticles, n ≥ 3.

Table S2. The accumulation of nanoparticles (%ID) in the form of heatmap table for passive (P) and magnetic (M) targeting. Cells are color-coded on a red-yellow-green scale, where red indicates the minimum value and green indicates the maximum value.

	Targeting	Liver	Spleen	Lungs	Kidney	Kidney	Heart	Muscle	Bone	Brain	Blood	Tumor
CMD 4 kDa	P	95.34	4.00	0.24	0.05	0.04	0.00	0.01	0.01	0.01	0.06	0.24
	M	87.73	9.39	0.93	0.13	0.07	0.01	0.03	0.01	0.01	0.08	1.61
CMD 10-20 kDa	P	88.36	10.44	0.51	0.09	0.06	0.01	0.02	0.07	0.02	0.08	0.36
	M	88.03	9.19	1.53	0.05	0.05	0.01	0.02	0.02	0.01	0.12	0.99
CMD 40 kDa	P	92.27	6.19	0.53	0.03	0.08	0.01	0.04	0.08	0.01	0.18	0.58
	M	92.71	5.32	1.04	0.05	0.05	0.01	0.01	0.01	0.01	0.08	0.71
CMD 70 kDa	P	90.39	6.60	1.72	0.04	0.04	0.01	0.09	0.17	0.03	0.13	0.87
	M	86.04	6.72	4.45	0.10	0.11	0.05	0.12	0.05	0.02	0.15	2.27
CMD 150 kDa	P	88.73	7.75	0.75	0.04	0.04	0.01	0.04	0.13	0.02	0.22	2.31
	M	85.44	6.71	3.71	0.12	0.13	0.03	0.14	0.26	0.02	0.97	2.48
Dext	P	92.86	5.66	0.86	0.04	0.04	0.01	0.06	0.01	0.04	0.15	0.28
	M	84.58	9.26	2.18	0.10	0.10	0.02	0.03	0.02	0.04	1.82	1.85
PVA	P	94.05	4.70	0.44	0.03	0.06	0.02	0.02	0.03	0.06	0.27	0.32
	M	91.99	4.96	1.55	0.06	0.06	0.03	0.03	0.02	0.05	0.38	0.87
PAA	P	91.41	6.89	0.36	0.08	0.08	0.03	0.05	0.05	0.10	0.44	0.51
	M	92.51	5.59	0.81	0.08	0.07	0.02	0.03	0.03	0.05	0.28	0.52
PVP	P	91.61	7.42	0.28	0.06	0.03	0.01	0.03	0.01	0.07	0.20	0.27
	M	86.56	9.69	1.15	0.10	0.06	0.02	0.05	0.02	0.03	0.23	2.09
PSSS 70 kDa	P	93.39	5.78	0.14	0.04	0.03	0.03	0.02	0.02	0.06	0.21	0.28
	M	88.87	7.57	1.47	0.08	0.06	0.02	0.14	0.04	0.05	0.16	1.54
PSSS 500 kDa	P	90.14	8.20	0.43	0.05	0.08	0.04	0.05	0.06	0.07	0.46	0.42
	M	85.41	11.93	0.57	0.10	0.10	0.04	0.10	0.06	0.10	0.27	1.32
Sod. Citrate	P	86.51	11.56	0.33	0.07	0.09	0.03	0.06	0.03	0.13	0.54	0.63
	M	85.59	11.07	1.50	0.10	0.07	0.05	0.07	0.03	0.05	0.22	1.26
ConA	P	87.04	11.53	0.57	0.05	0.05	0.01	0.02	0.03	0.12	0.21	0.36
	M	69.92	11.58	16.13	0.23	0.23	0.08	0.04	0.04	0.06	0.61	1.07
SBA	P	84.17	14.31	0.54	0.06	0.07	0.03	0.04	0.07	0.06	0.21	0.44
	M	84.04	12.33	1.39	0.09	0.06	0.04	0.05	0.06	0.06	0.17	1.71
PEI 50 mg	P	34.22	15.03	47.63	0.43	0.39	0.42	0.04	0.14	0.07	0.59	1.04
	M	20.19	8.12	69.28	0.44	0.39	0.42	0.05	0.02	0.07	0.59	0.43
PEI 100 mg	P	39.73	14.19	40.95	0.84	0.83	0.76	0.11	0.22	0.14	0.63	1.60
	M	19.09	5.59	71.75	0.70	0.75	0.67	0.07	0.04	0.22	0.63	0.49
Chit	P	80.86	17.37	1.01	0.07	0.03	0.01	0.03	0.02	0.14	0.19	0.27
	M	54.69	15.28	26.77	0.37	0.31	0.15	0.05	0.06	0.09	0.52	1.71
Uncoated magnetite	P	81.06	17.55	0.56	0.12	0.06	0.03	0.05	0.02	0.03	0.21	0.29
	M	78.12	13.36	6.52	0.12	0.10	0.02	0.04	0.02	0.03	0.15	1.51

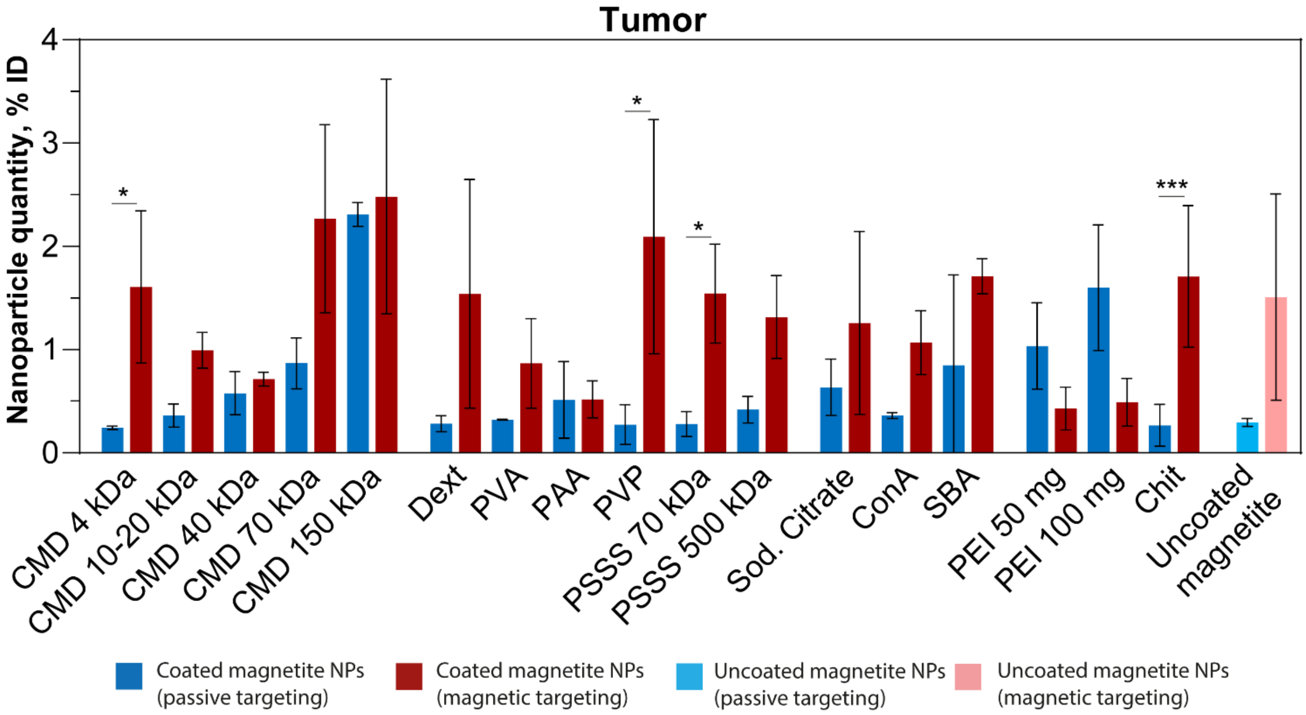

Figure S6. Tumor accumulation of coated and uncoated magnetite nanoparticles. Asterisks indicate significant differences between tumor accumulation percentages of NPs with passive and magnetic targeting, determined by two-tailed Welch's t-tests; * – $p \leq 0.05$; ** – $p \leq 0.01$; *** – $p \leq 0.001$; $n \geq 3$.

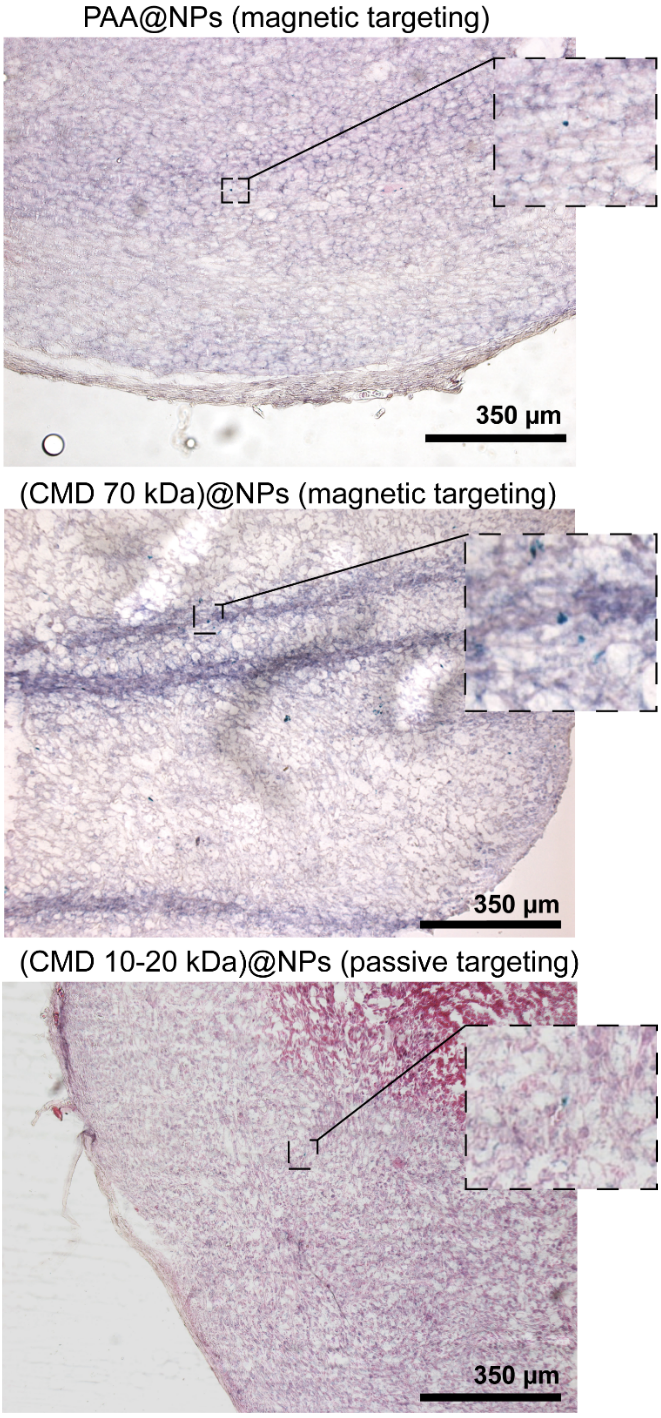

Figure S7. Representative histological sections of tissues from mice injected with different nanoparticles under various targeting conditions.

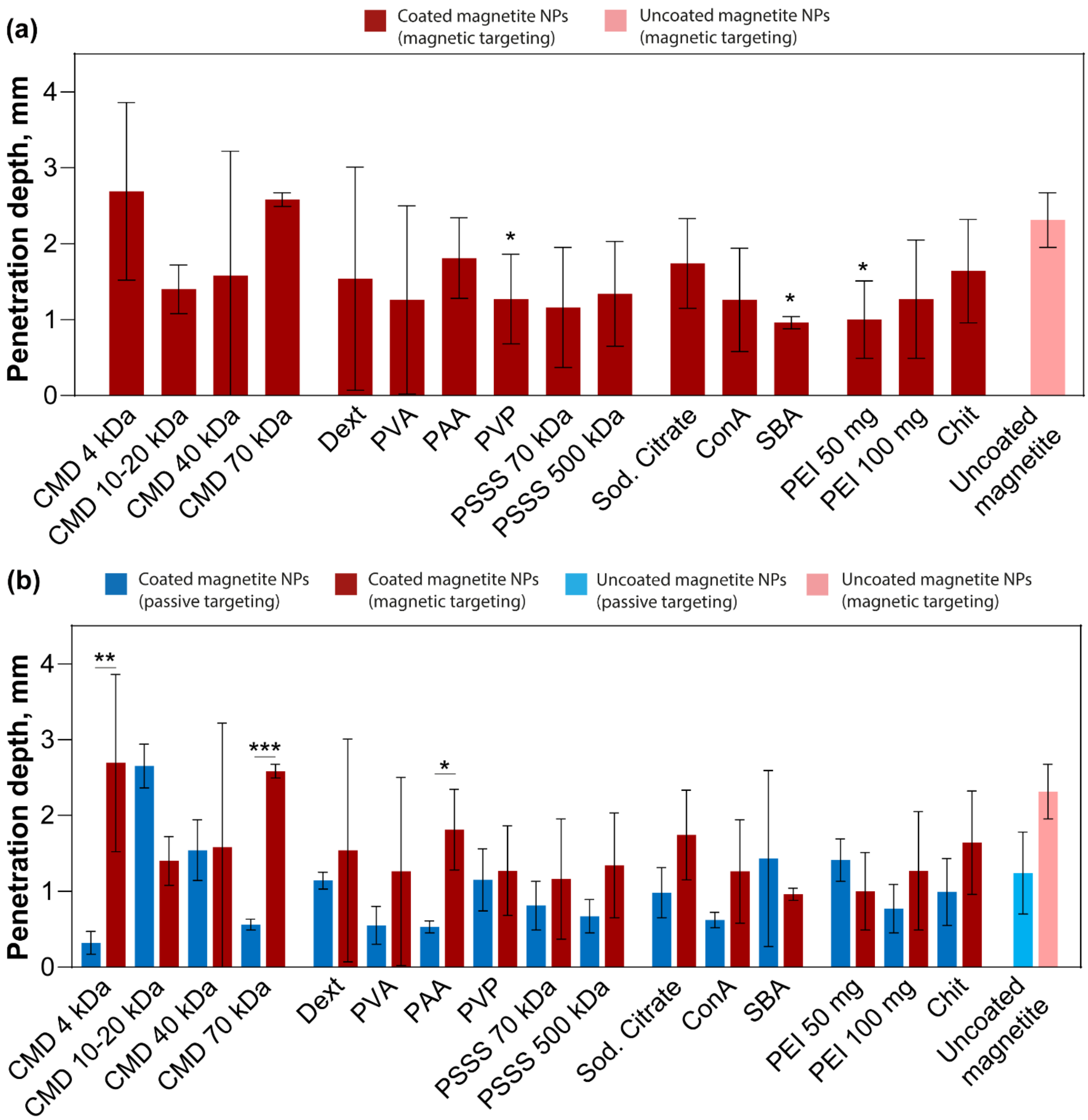

Figure S8. Statistically significant difference in tumor tissue penetration depth (a) between coated and uncoated magnetite nanoparticles with magnetic targeting; (b) between passive and magnetic targeting for coated and uncoated magnetite nanoparticles. Asterisks indicate significant differences determined by two-tailed Welch's t-tests; * – $p \leq 0.05$; ** – $p \leq 0.01$; *** – $p \leq 0.001$; $n \geq 3$.
